# Rapid Response Fluorescence Probe Enabled In Vivo Diagnosis and Assessing Treatment Response of Hypochlorous Acid‐Mediated Rheumatoid Arthritis

**DOI:** 10.1002/advs.201800397

**Published:** 2018-06-07

**Authors:** Huan Feng, Zhiqiang Zhang, Qingtao Meng, Hongmin Jia, Yue Wang, Run Zhang

**Affiliations:** ^1^ School of Chemical Engineering University of Science and Technology Liaoning Anshan Liaoning 114051 P. R. China; ^2^ Australian Institute for Bioengineering and Nanotechnology The University of Queensland Brisbane 4072 Australia

**Keywords:** fluorescence probes, hypochlorous acid, inflammatory diseases, rheumatoid arthritis, treatment response

## Abstract

Diagnosis and early assessment of the treatment response of rheumatoid arthritis (RA) necessitates a reliable bioanalytical method for rapid, sensitive, and specific detection of the hypochlorous acid (HOCl) biomarker in inflammatory diseases. Herein, two fluorescence probes, Probe‐**1** and Probe‐**2** are developed for quantitative monitoring and visualization of inflammatory response–related HOCl levels in vitro and in vivo. In the presence of HOCl, fluorescence “OFF–ON” response is obtained for both the probes as a result of specific HOCl‐triggered C=N bond cleavage reaction. Probe‐**1** and Probe‐**2** feature rapid response (<4 s), a high degree of sensitivity and selectivity toward HOCl, which allow them to be used for quantification of HOCl in a simulated physiological condition. Using Probe‐**2** as the probe, fluorescence imaging and flow cytometry analysis of HOCl levels in lysosome of inflammatory mimic cells, visualization of HOCl generation in endotoxin‐induced inflammation of adult zebrafish and RA of mice are possible. Probe‐**2** exhibits high effectiveness for early assessment of the treatment response of HOCl‐mediated RA in mice with an antiarthritic drug, methotrexate (MTX). The results demonstrate that Probe‐**2** is a powerful tool for future studies on diagnosis and monitoring treatment efficiency in a broad range of inflammatory diseases, including RA.

## 1 Introduction

Rheumatoid arthritis (RA) is a chronic autoimmune inflammatory disorder, characterized by intense inflammatory and immunological reaction mainly in peripheral joints.^1^ This disease affects ≈25 million people, ≈1% of adults in the world population.^2^ It is reported that genetic and environmental factors maybe the cause for RA, but the etiology for this inflammatory disease is not fully elucidated.^3^ For the treatment of RA, low dose of methotrexate (MTX) has long been recognized as a standard for patients suffering from this disease,^4^ yet the mechanism involved in its activity against RA remains less clear.^1^, ^5^ Recently, studies showed that the elevated reactive oxygen species (ROS),^6^ in particular an overabundance of hypochlorous acid (HOCl) generation is implicated in the pathogenesis of various inflammatory disorders,^7^ i.e., increasing levels of HOCl generation might be recognized as one of hallmark for inflammatory chronic arthropathies. Therefore, the development of effective bioanalytical method for rapid, sensitive, and specific detection of HOCl in inflammatory mimic cells and organisms will significantly contribute to better understand the roles of this molecule in RA and also benefit to the assessing of the treatment response of RA.

HOCl is generated endogenously in living organisms by the reaction of H_2_O_2_ with Cl^−^ ions under the catalysis of a heme enzyme, myeloperoxidase (MPO).^8^ It is reported that the diffusion distance of HOCl is less than 20 µm.^9^ Within such a distance, the endogenous HOCl can rapidly react with other biomolecules and coexisting antioxidants, such as glutathione (GSH), cysteine (Cys), and ascorbic acid, thus leading to extreme challenges in tracking this molecule in situ and in vivo.[[qv: 7,8c,10]] Toward this end, a number of bioanalytical methods, such as colorimetric, bioluminescent, luminescent/fluorescent, electrochemical, and chromatographic methods, have been reported in the past decades.^11^ Of these methods, fluorescent probes have been proven to be an indispensable tool for real‐time visualization and analysis of the localization and dynamics metabolism of HOCl in biological systems, owing to their versatile advantages such as high sensitivity, simplicity for implementation, real‐time detection, and good compatibility for biological samples.^12^ For many years, we have also focused on the development of novel bioanalytical methods for HOCl detection in biological systems, especially the HOCl generated in inflammatory diseases, such as drug‐induced liver injury and ischemia–reperfusion (I/R) injury.^13^ As part of our ongoing research, we recently focused on engineering rapid and effective HOCl responsive probes for unveiling the detailed roles of HOCl in RA diagnosis and further evaluating the treatment response of RA by antiarthritic drug.

**Scheme 1:**
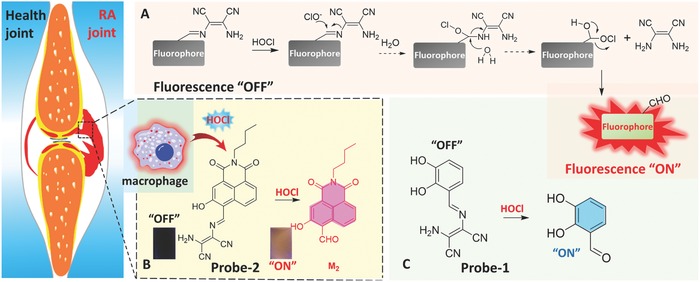
A) Schematic illustration of the design strategy and the sensing mechanism of the probes for monitoring of HOCl‐mediated RA. Chemical structure of B) Probe‐**2** and C) Probe‐**1** and its reaction with HOCl.

In this contribution, two fluorescence probes, Probe‐**1** and Probe‐**2** were designed and synthesized for in situ visualization and analysis of the HOCl in inflammatory disorder (**Scheme** **1**). Both the probes were synthesized through a one‐step condensation reaction, and the chemical structure and molecular weight of probes were well characterized by high‐resolution mass spectrometry (HRMS), NMR, and Fourier transform infrared spectroscopy (FTIR). Probe‐**1** and Probe‐**2** exhibited extremely weak fluorescence due to the C=N bond isomerization, while the fluorescence emission and absorption properties can be significantly changed in the presence of HOCl. This sensing mechanism for both Probe‐**1** and Probe‐**2**, i.e., HOCl‐triggered C=N bond cleavage reaction,^7^, ^14^ was confirmed by HRMS titration. In the presence of HOCl, fluorescence “OFF–ON” response was found to be completed within 3 s for Probe‐**1** and within 4 s for Probe‐**2**. Such a rapid fluorescence response of probes enables real‐time monitoring of the HOCl generation in biological systems. Of these two probes, Probe‐**2** featured fast response to HOCl, high sensitivity and specificity, superb excitation/emission wavelength, and biocompatibility in both HeLa cells and J774A.1 macrophage cells. With such a probe in hand, the HOCl‐mediated inflammatory response in living cells was investigated by fluorescence microscopy imaging and flow cytometry analysis. Fluorescence imaging revealed the lysosome distribution of Probe‐**2** in J774A.1 macrophage cells, which allowed HOCl detection in inflammatory mimic cells at subcellular level. The results of visualization of drug‐induced inflammatory reaction in adult zebrafish, RA of mice, and HOCl‐mediated treatment response of RA imply that Probe‐**2** holds promise for future diagnosis and assessing treatment response of inflammatory diseases such as RA.

## 2 Results and Discussion

### 2.1 Design, Synthesis, and Characterization of Fluorescence Probes

Considering the important roles of HOCl in inflammatory disease of living organisms, Probe‐**1** and Probe‐**2** were designed and synthesized for rapid, sensitive, and selective detection of HOCl in aqueous solution, inflammatory response in living cells, zebrafish, and mice. As shown in Scheme 1, Probe‐**1** and Probe‐**2** were designed by linking a 2,3‐diaminomaleonitrile to fluorophore through a C=N bond. The probes are nonfluorescent because decay processes of their excited states are perturbed by the C=N isomerization.^15^ Therefore, the fluorescence emission of both Probe‐**1** and Probe‐**2** are quenched. In the presence of HOCl, the C=N bond can be specifically cleaved to form the aldehyde–fluorophore derivatives (**M_1_**, **M_2_**), thereby leading to strong fluorescence emission. The concentration of HOCl is thus being determined by recording the changes of fluorescence emission. With such “OFF–ON” responsive fluorescence probes, we envisioned that the HOCl‐mediated inflammatory response and RA treatment response could be revealed.

As shown in Scheme S1 (Supporting Information), Probe‐**1** and Probe‐**2** were prepared by a one‐step condensation reaction between 2,3‐diaminomaleonitrile and 2,3‐dihydroxbenealdehyde (**M_1_**), 4‐formyl‐3‐hydroxy‐1,8‐naphthalic‐*n*‐butylimide (**M_2_**), respectively. The structures of both the probes were confirmed by ^1^H NMR, ^13^C NMR, 2D NMR, HRMS, and FTIR spectra (Figures S1–S12, Supporting Information). To confirm the fluorescence response mechanism of probes toward HOCl shown in Scheme 1, the solutions of the reactions between HOCl and Probe‐**1** and Probe‐**2** were analyzed by HRMS. For Probe‐**1**, HRMS analysis exhibited a molecular ion peak at *m*/*z* = 137.0259 (Figure S13, Supporting Information), which can be assigned to the peak of [**M_1_**—H]^−^ (Calcd. *m*/*z* = 137.0244). Similarly, a molecular ion peak at *m*/*z* = 296.0924 was found for the solution containing Probe‐**2** and HOCl (Figure S14, Supporting Information), which can be ascribed to the peak of [**M_2_**—H]^−^ (Calcd. *m*/*z* = 296.0928). The HRMS analysis results indicated that the unbridged C=N bonds of Probe‐**1** and Probe‐**2** have been successfully cleaved by a specific reaction with HOCl, leading to formation of the corresponding aldehyde–fluorophore derivatives.

### 2.2 UV–Vis Spectra Responses of Probe‐**1** and Probe‐**2** toward HOCl

**Figure 1:**
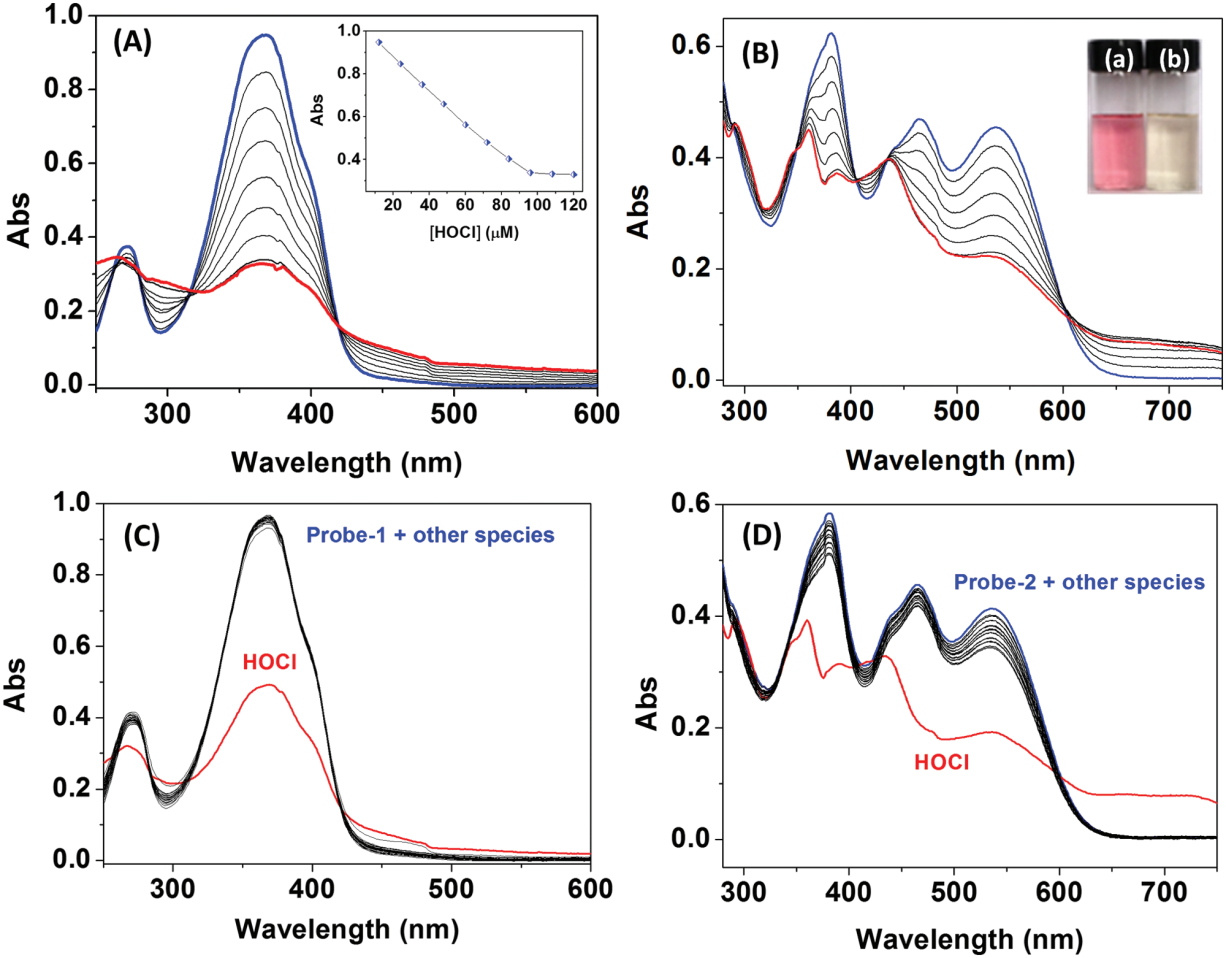
A) UV–vis absorption spectra of Probe‐**1** (10 × 10^−6^ m) in PBS buffer (DMSO:H_2_O = 3:7, v/v; pH 7.4) in the presence of different concentrations of HOCl (0–120 × 10^−6^ m), inset: plot of the absorbance at 367 nm of Probe‐**1** against the concentration of HOCl. B) UV–vis absorption spectra of Probe‐**2** (10 × 10^−6^ m) in PBS buffer (DMSO:H_2_O = 3:7, v/v; pH 7.4) upon the addition of different concentrations of HOCl (0–100 × 10^−6^ m), inset: the color change of Probe‐**2** in the presence of HOCl. C) UV–vis absorption spectra of Probe‐**1** (10 × 10^−6^ m) in PBS buffer (DMSO:H_2_O = 3:7, v/v; pH 7.4) in the presence of various ROS and anion species (120 × 10^−6^ m). D) UV–vis absorption spectra of Probe‐**2** (10 × 10^−6^ m) in PBS buffer (DMSO:H_2_O = 3:7, v/v; pH 7.4) in the presence of various ROS and anion species (100 × 10^−6^ m).

UV–vis absorption responses of Probe‐**1** (10 × 10^−6^ m) and Probe‐**2** (10 × 10^−6^ m) toward HOCl were first investigated in phosphate buffered solution (PBS) buffer of pH 7.4. As shown in **Figure** **1**A, the UV–vis absorption spectrum of Probe‐**1** exhibits two strong absorption bands centered at 271 and 367 nm, which can be ascribed to the typical intramolecular charge transfer (ICT)–dominated absorption process.^16^ Upon addition of HOCl at the concentration of 0–120 × 10^−6^ m, the maximum absorption band at 367 nm of Probe‐**1** was gradually diminished. The changes of absorption at 367 nm could be assigned to the disruption of the ICT process by a specific HOCl‐triggered C=N bond cleavage reaction.[[qv: 7,12h,14c]] Figure 1B illustrated the changes of UV–vis absorption spectra of Probe‐**2** (10 × 10^−6^ m) in the presence of different concentrations of HOCl (0–100 × 10^−6^ m) in PBS buffer (dimethyl sulfoxide (DMSO):H_2_O = 3:7, v/v; pH 7.4). Similarly, the absorbance bands of Probe‐**2** centered at 381, 464, and 536 nm were gradually decreased under a HOCl‐triggered C=N bond cleavage reaction. Accordingly, the color of the Probe‐**2** solution was changed from pink to pale yellow after addition of 10.0 equiv of HOCl (Figure 1B inset), suggesting that Probe‐**2** can serve as a potential indicator for “naked eye” detection of HOCl in water samples.

To evaluate the selectivity of Probe‐**1** and Probe‐**2** toward HOCl, the changes of absorption spectra were then investigated in the presence of various ROS and anion species, and the results were illustrated in Figure 1C,D. As expected, no obvious changes of absorption spectra were noticed upon the addition of competitive species, including Cl^−^, Br^−^, I^−^, SO_4_^2−^, HCO_3_^−^, PO_4_^3−^, Pi, NO_3_^−^, NO_2_^−^, ^1^O_2_, H_2_O_2_, ·OH, and ONOO^−^, demonstrating that Probe‐**1** and Probe‐**2** are highly selective toward HOCl over other ROS and anions. The specific UV–vis response of Probe‐**2** toward HOCl was also confirmed by colorimetric assay (Figure S15, Supporting Information), where the significant changes in color occurred in the presence of HOCl over other species.

### 2.3 Fluorescence Response of Probe‐**1** and Probe‐**2** toward HOCl

**Figure 2:**
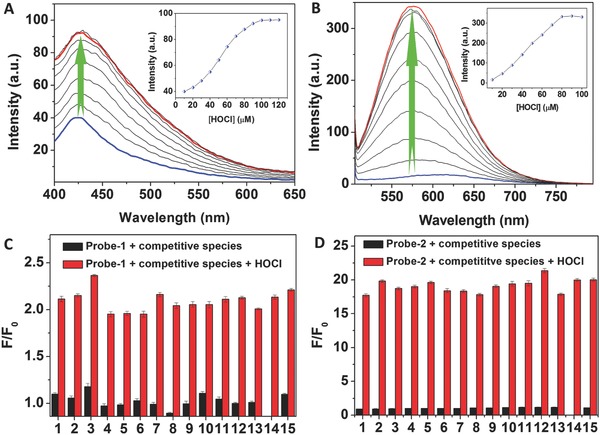
Fluorescence response of Probe‐**1** and Probe‐**2** toward HOCl. Fluorescence spectra of A) Probe‐**1** (10 × 10^−6^ m) and B) Probe‐**2** (10 × 10^−6^ m) in PBS buffer (DMSO:H_2_O = 3:7, v/v; pH 7.4) upon the addition of increasing amounts of HOCl. Enhanced fluorescence intensity factor (*F*/*F*_0_) of C) Probe‐**1** emission at 427 nm and D) Probe‐**2** emission at 575 nm in the presence of various ROS and anion species in PBS buffer (DMSO:H_2_O = 3:7, v/v; pH 7.4). The competitive species include: (1) Cl^−^, (2) Br^−^, (3) I^−^, (4) SO_4_^2−^, (5) HCO_3_^−^, (6) PO_4_^3−^, (7) inorganic phosphates (Pi), (8) NO_3_^−^, (9) NO_2_^−^, (10) ^1^O_2_, (11) H_2_O_2_, (12) ·OH, (13) ONOO^−^, (14) HOCl, and (15) mixed species. Excitations were performed at 370 and 490 nm for Probe‐**1** and Probe‐**2**, respectively.

The fluorescence responses of Probe‐**1** and Probe‐**2** toward HOCl were then evaluated by spectrometric titration in PBS buffer (DMSO:H_2_O = 3:7, v/v; pH 7.4). As shown in **Figure** **2**A,B, Probe‐**1** and Probe‐**2** exhibited weak fluorescence emission under excitation at 370 and 490 nm, respectively. Upon addition of HOCl, the fluorescence intensities for both the probes were significantly increased. The enhancement in fluorescence emission can be attributed to the formation of **M_1_** and **M_2_** after a HOCl‐triggered C=N bond cleavage reaction.^17^ The maximum enhancement in fluorescence intensity was obtained after addition of 10 equiv of HOCl to Probe‐**1** and 8 equiv of HOCl to Probe‐**2**. Using fluorescein as the reference,^18^ the quantum yields for Probe‐**1** and Probe‐**2** were measured to be *Φ*_1_ = 0.0423 ± 0.0041 and *Φ*_2_ = 0.0106 ± 0.0008, respectively. In the presence of HOCl (12.0 equiv for Probe‐**1** and 10.0 equiv for Probe‐**2**), the quantum yields of the products (**M_1_** and **M_2_**) were determined to be *Φ*_3_ = 0.177 and *Φ*_4_ = 0.333, respectively. As shown in Figure S16 (Supporting Information), the changes of fluorescence intensity of Probe‐**1** and Probe‐**2** exhibited good linearity toward the concentration of HOCl. The detection limit (LOD) was then calculated according to the concentration corresponding to three standard deviations of the background signal (LOD = 3σ/*k*).^19^ Accordingly, the LOD for HOCl detection were determined to be 208.9 × 10^−9^ and 17.3 × 10^−9^ m for Probe‐**1** and Probe‐**2**, respectively. Clearly, Probe‐**2** showed higher sensitivity, which enables it to be further used as the fluorescence probe for visualization of HOCl‐mediated inflammatory response in vitro and in vivo.

To evaluate the selectivity of Probe**‐1** and Probe‐**2** toward HOCl, the changes of fluorescence intensity for both the probes toward various analytes were measured and the results were presented in Figure 2C,D. A significant increase in fluorescence intensity of Probe‐**1** at 427 nm was observed in the presence of HOCl, while negligible changes of fluorescence intensity were obtained upon addition of other species, including Cl^−^, Br^−^, I^−^, SO_4_^2−^, HCO_3_^−^, PO_4_^3−^, Pi, NO_3_^−^, NO_2_^−^, ^1^O_2_, H_2_O_2_, ·OH, and ONOO^−^. Similarly, Probe‐**2** also showed no response in fluorescence toward other ROS and anions, which is obviously different from the result of the addition of HOCl. The results suggested the high selectivity of Probe‐**2** toward HOCl detection. Specific fluorescence response of Probe‐**2** toward HOCl can also be supported by “naked eye” analysis, where the changes of fluorescence color for each mixture solution were recorded (Figure S17, Supporting Information). The effects of biological cations and biothiols to the fluorescence detection of HOCl were then investigated by addition of cations, including Li^+^, Na^+^, K^+^, Ca^2+^, Mg^2+^, Ba^2+^, Al^3+^, Fe^3+^, Cr^3+^, Zn^2+^, Co^2+^, Mn^2+^, Cu^2+^, Cys, Hcy, and GSH. As shown in Figure S18 (Supporting Information), no significant enhancement in fluorescence intensity was noticed for both Probe‐**1** and Probe‐**2**. In addition, in the presence of various amino acids, the effects of fluorescence intensity of **M_1_** and **M_2_** (the products of Probe‐**1** and Probe‐**2** reacted with HOCl, respectively) were evaluated. As shown in Figure S19 (Supporting Information), no obvious changes of fluorescence intensities for both the probes were obtained. The results indicated that Probe‐**1** and Probe‐**2** can be employed as the probe specific for HOCl detection under physiological condition.

**Figure 3:**
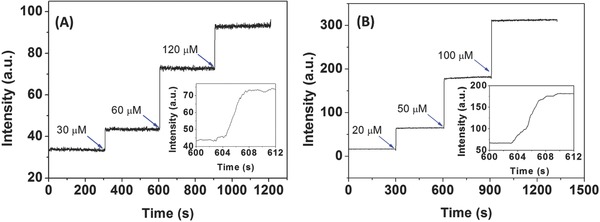
A,B) Fluorescence enhancement time profiles of Probe‐**1** (10 × 10^−6^ m) and Probe‐**2** (10 × 10^−6^ m) for the addition of HOCl in PBS buffer (DMSO:H_2_O = 3:7, 20 × 10^−3^ m, pH 7.4). Inset: time courses of fluorescence intensity changes of Probe‐**1** (10 × 10^−6^ m) and Probe‐**2** (10 × 10^−6^ m) after addition of HOCl within seconds. The excitation and emission wavelength (λ_ex_/λ_em_) are 370/427, 490/575 nm for Probe‐**1** and Probe‐**2**, respectively.

It is well documented that the short‐lived HOCl is highly reactive toward biomolecules inside the body.^20^ The probe with the ability of rapid fluorescence response toward HOCl is the key to monitor this mediator in biological samples. Therefore, time‐profile fluorescence responses of Probe‐**1** and Probe‐**2** toward HOCl were examined and the results were presented in **Figure** **3**. Upon light irradiation, both Probe‐**1** and Probe‐**2** exhibited weak and stable fluorescence emission in the absence of HOCl. After addition of HOCl, the fluorescence intensity was rapidly increased. The fluorescence intensity reached to the maximum level within a few seconds (3 s for Probe‐**1** and 4 s for Probe‐**2**). Upon another amount of HOCl addition, the fluorescence intensity was rapidly increased again and reached to another maximum level. As shown in Figure S20 (Supporting Information), HOCl at different concentrations was added into the solution containing Probe‐**1** or Probe‐**2**, and the changes of fluorescence intensities were recorded. A good linearity between the HOCl concentration and the corresponding initial reaction rate is obtained. Following the reported methods,^21^ the total reaction rate constants (*k*_tot_) of the Probe‐**1**/HOCl and Probe‐**2**/HOCl reaction in PBS buffer were calculated to be 3.54 × 10^10^ and 3.35 × 10^10^ m ^−1^ s^−1^, respectively. The results demonstrated that the reactions between probes and HOCl are very fast and the reaction products are stable in PBS buffer.

The effects of pH on the C=N cleavage reaction between Probe‐**1**, Probe‐**2**, and HOCl were evaluated (DMSO:H_2_O = 3:7, v/v) with different pH levels. As shown in Figure S21 (Supporting Information), the fluorescence intensity of Probe‐**1** and Probe‐**2** showed weak and stable emissions at different pHs ranging from 4.5 to 11.0. In the presence of HOCl, the fluorescence intensities of both probes were significantly increased at all pHs. To examine the stability of C=N bond of probe, the changes of fluorescence intensity of Probe‐**2** were then evaluated in the buffers of pH 4–5. As shown in Figure S22 (Supporting Information), very small enhancements in fluorescence intensity at 575 nm were observed for the solution of Probe‐**2** in buffer of pH 4, 4.5, and 5. By contrast, upon addition of HOCl, significant enhancements in fluorescence intensity at 575 nm were noticed immediately for the solution of Probe‐**2** in buffer of pH 4, 4.5, and 5. The results indicated that both Probe‐**1** and Probe‐**2** can work well as the fluorescence probe for the detection of HOCl in weakly acidic, neutral, and weakly basic buffers.

### 2.4 HOCl‐Mediated Inflammatory Response in Living Cells

Considering the excitation/emission wavelength and the sensitivity of the probes, Probe‐**2** was selected as the fluorescence probe for quantitative detection of HOCl in biological samples, i.e., analysis of HOCl‐mediated inflammatory responses in living cells and organisms. The cytotoxicity of Probe‐**2** in HeLa and J774A.1 macrophage was initially examined. HeLa cells were incubated with Probe‐**2** at the concentration of 0, 2 × 10^−6^, 5 × 10^−6^, 8 × 10^−6^, 10 × 10^−6^, and 20 × 10^−6^ m for 24 h, and then treated with 3‐(4,5‐dimethylthiazol‐2‐yl)‐2,5‐diphenyltetrazolium bromide (MTT) for 4 h. As shown in Figure S23 (Supporting Information), the HeLa cell viability was found to be greater than 90% even after incubation of Probe‐**2** for 24 h at the concentration of 20 × 10^−6^ m. For investigation of the cytotoxicity of Probe‐**2** in J774A.1 macrophage cells, the cells were treated with Probe‐**2** at the concentration of 0, 2 × 10^−6^, 5 × 10^−6^, 8 × 10^−6^, 10 × 10^−6^, and 20 × 10^−6^ m for 24 h, followed by the incubation with PrestoBlue for 10 min before measuring the emission intensity at 600 nm. The cell viability remains greater than 80% after incubation of Probe‐**2** for 24 h at the concentration of 10 × 10^−6^ m, while the cell viability is reduced to be 54% at the concentration of 20 × 10^−6^ m. For the detection of HOCl in biological samples, the concentration of Probe‐**2** was 4 × 10^−6^ m for living cell imaging and flow cytometry analysis, and 10 × 10^−6^ m for in vivo experiments in this work. Therefore, at such low concentration of Probe‐**2**, the effects of this probe toward living cells and organisms can be ignored as all experiments that can be completed within 1.5 h.

The cytotoxicity of **M_2_** toward HeLa cells and J774A.1 macrophage cells was then evaluated by MTT assay and PrestoBlue viability assay, respectively. As shown in Figure S23 (Supporting Information), no obvious cytotoxicity of **M_2_** to HeLa cells was obtained as the cell viability was more than 90% even after treating the HeLa cells with 20 × 10^−6^ m **M_2_** for 24 h. For the cytotoxicity of J774A.1 macrophage cells, the results showed that the cell viability was kept greater than 89% for the cells treated with 10 × 10^−6^ m **M_2_** for 24 h. When the concentration of the **M_2_** increased to 20 × 10^−6^ m, the cell viability was found to be more than 75% after 24 h coincubation. These results indicated that the product of the reaction between Probe‐**2** and HOCl, **M_2_** is also of low cytotoxicity.

To further study the cell permeability, the lipophilicity (the key criteria for the uptake of the probes) of Probe‐**2** was evaluated following reported methods.^22^ By measuring the partition coefficient (log*P*_o/w_) between 1‐octanol and water, the lipophilicity of Probe‐**2** was determined to be 1.27. It is well known that the log*P*_o/w_ value of a fluorescence probe located within the range of 0–5 shows good cellular membrane permeability. Therefore, we reasoned that Probe‐**2** is able to permeate into cells easily.

The proof‐of‐concept experiment for analysis of HOCl in biological samples was first conducted through imaging of exogenous HOCl in living HeLa cells. The cells were incubated with Probe‐**2** (4 × 10^−6^ m) for 30 min, followed by the treatment with HOCl (10 × 10^−6^ m) for another 15 min. As shown in Figure S24 (Supporting Information), Probe‐**2**‐deposited HeLa cells exhibited weak fluorescence, while the intracellular fluorescence was significantly increased after further incubation of the cells with HOCl for 15 min. These experiments suggested that Probe‐**2** is cell membrane permeable, and that the HOCl in living cells can be detected using Probe‐**2** as the fluorescence probe.

Quantitative detection of intracellular HOCl in single HeLa cells was then confirmed by flow cytometry analysis, where the fluorescence intensities of 10 000 cells were measured from each cell population. The shifts of the histogram and the mean fluorescence intensity (MFI) of HeLa cells were measured and the results were illustrated in Figures S25 and S26 (Supporting Information). Both the control groups (HeLa cells and Probe‐**2**‐stained HeLa cells) showed low background fluorescence emission, while obvious shift of the histogram to the direction of strong fluorescence was noticed after further incubation the Probe‐**2**‐stained HeLa cells with HOCl. Quantitative detection of HOCl in a single HeLa cell was also achieved by recording the MFI of each cell population (Figure S26, Supporting Information). Significant enhancement in fluorescence intensity of each cell population was observed, indicating that Probe‐**2** can be used as the fluorescence probe for quantitative detection of intracellular HOCl at single cell level.

**Figure 4:**
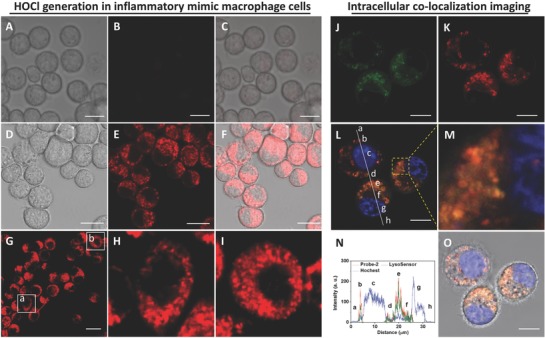
Bright‐field, fluorescence, and merged images of endogenous HOCl generation in LPS‐induced inflammatory mimic J774A.1 macrophage. A–C) The macrophage was incubated with 4 × 10^−6^ m Probe‐**2** for 30 min. D–F) J774.1 macrophage cells were treated with LPS (1.0 µg mL^−1^) for 4 h, and then incubated with Probe‐**2** for another 30 min. G) Fluorescence imaging of endogenous HOCl generation in macrophage cells with LPS stimulation. H,I) The cells of interest shown in (a) and (b) of (G). J–O) Intracellular colocalization analysis of Probe‐**2** with LysoSensor Green in living J774A.1 macrophage cells. (J) The lysosome of J774A.1 cells was stained by LysoSensor Green. (K) Cells were stimulated with LPS (1.0 µg mL^−1^) for 4 h, and then incubated with Probe‐**2** for another 30 min. (L) Merged imaging of (J), (K), and the cell nucleus stained with Hochest 33342, the area of interest (a, d, h): intercellular space, (b, e): lysosome, (d, g): cell nucleus, (f): cytoplasm. (M) Zoom‐in fluorescence image of selected area in (L). (N) Fluorescence intensity profiles of the linear region of interest across macrophage cells in (L). (O) Merged image of (L) and corresponding bright‐field image. Scale bars are 20 µm.

Having confirmed the desirable performance of Probe‐**2** for the detection and imaging of HOCl in living cells, we moved on to investigate its ability to visualize HOCl‐mediated inflammatory response in living macrophage cells. Inflammatory mimic J774A.1 macrophage cells were obtained by lipopolysaccharide (LPS) stimulation.^23^ After 4 h, the cells were incubated with Probe‐**2** for 30 min. J774A.1 macrophage cells without stimulation were employed as the control group. As shown in **Figure** **4**A–C, Probe‐**2**‐loaded macrophage cells exhibited dark fluorescence, while bright fluorescence was noticed for the inflammatory mimic cells, showing that pretreatment with LPS was performed (Figure 4D–F). The results of macrophage cells imaging demonstrated that the Probe‐**2** was sensitive enough for monitoring the HOCl‐mediated inflammatory response in living macrophage cells.

As shown in Figure 4G–I, the microscope images of macrophage cells clearly indicated that the intracellular fluorescence was presented in isolated spherical vesicles in the cytoplasm. This result promoted us to study the intracellular distribution of these spherical vesicles by colocalization experiment, where the cells were stained with both Probe‐**2** and lysosome sensor, LysoSensor Green. As shown in Figure 4J–O and Figure S27 (Supporting Information), obvious overlap between red fluorescence from Probe‐**2** and green fluorescence from LysoSensor Green was obtained. Subcellular regions, such as lysosome (b, e), cell nucleus (c, g), and cytoplasm (f) can be clearly distinguished (Figure 4L,N). The fluorescence intensity profiles of linear regions of interest across J774A.1 macrophage cells that were stained with both Probe‐**2** and LysoSensor Green vary in close synchrony (Figure 4N and Figure S27H (Supporting Information)). The Pearson's correlation coefficient and Mander's overlap coefficient were determined to be 0.945 and 0.970, respectively. Both coefficients are close to 1 in cell images, suggesting high colocalization of Probe‐**2** and LysoSensor Green in J774A.1 macrophage cells.^24^ The intensity correlation analysis was then performed to investigate the fluorescence intensity distribution of the Probe‐**2** and LysoSensor Green (Figure S27, Supporting Information). The intensity correlation quotient (ICQ) was determined to be 0.411, which is very close to 0.5, suggesting that the stains of Probe‐**2** and LysoSensor Green are dependent. The above results suggested that Probe‐**2** can be used as the fluorescence probe for visualization of HOCl‐mediated inflammatory response at subcellular level.

### 2.5 HOCl Production in Inflammatory Response of Adult Zebrafish

**Figure 5:**
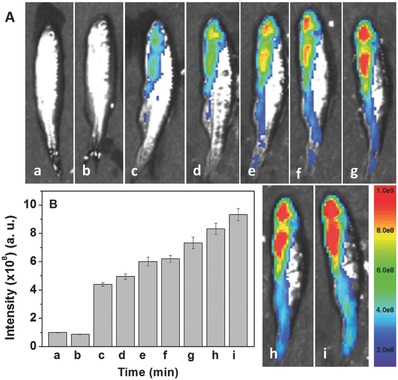
A) Fluorescence imaging of endogenous HOCl production in zebrafish. Zebrafish only (a); zebrafish was stimulated with LPS (2 µg mL^−1^) for 3 h (b); then stained with Probe‐**2** (10 × 10^−6^ m) for 1 min (c); 17 min (d); 34 min (e); 51 min (f); 78 min (g); 108 min (h); and 138 min (i), respectively. B) Mean fluorescence intensity of zebrafish treated at different conditions shown in (A).

With the promising living cell imaging data in hand, we were interested in the HOCl production in inflammatory response using adult zebrafish as a model.^25^ Apart from the LPS‐stimulated inflammation in living macrophage cells, LPS‐induced inflammatory response in zebrafish has long been recognized as an excellent model for biomedical researches on inflammatory disorders. Zebrafish was stimulated with LPS (2 µg mL^−1^) for 3 h, followed by the staining with Probe‐**2**, and then the images were recorded at different times. The zebrafish and the LPS‐stimulated zebrafish were employed as the control group. As shown in **Figure** **5**, no fluorescence was observed for the control groups, but clear fluorescence was observed for the LPS‐stimulated zebrafish that was coincubated with Probe‐**2**. The mean fluorescence intensity of zebrafish was increased over the time. The results indicated that the LPS‐induced inflammatory response in zebrafish can be visualized using Probe‐**2** as a fluorescence probe.

### 2.6 HOCl‐Mediated Inflammatory Response in RA of Mice

A vast amount of circumstantial evidence indicates that endogenous ROS production is one of the most important mediator of inflammation and/or tissue destruction in rheumatoid arthritis.^26^ With the observation of HOCl generation in inflammatory mimic macrophage cells, together with the result of HOCl‐mediated inflammation in adult zebrafish, we were then interested in investigating the roles of HOCl in inflammatory response of RA in mice, and evaluating the HOCl‐mediated RA treatment response by administering of antiarthritic drug. Prior to the imaging of endogenous HOCl generation in RA of mice, the ability of Probe‐**2** for visualization of exogenous HOCl in living mice was demonstrated. Probe‐**2** was subcutaneously injected into 6–8 week old nude mice, followed by the injection of HOCl at the same region. Fluorescence images were recorded at different time courses after injection. As shown in Figure S28 (Supporting Information), no fluorescence signal was observed in the absence of HOCl, whereas the fluorescence signal was recorded in the experimental groups, showing that the exogenous HOCl injection was applied. The fluorescence intensity was found to be gradually increasing within 20 min, and then was kept at the maximum intensity for at least 10 min (Figure S28i, Supporting Information). The result indicated that Probe‐**2** can be used as the fluorescence probe for imaging of HOCl in living mice.

**Figure 6:**
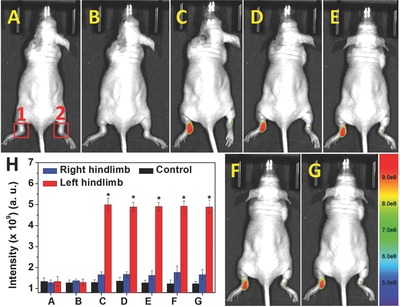
Visualization of HOCl‐mediated inflammatory response in RA of mice. A) Control group 1 (mice only); B) group 2 (left hind limbs were stimulated with λ‐carrageenan in PBS for 4 h); the Probe‐**2** was then injected and then the mice were imaged after C) 1 min, D) 5 min, E) 10 min, F) 15 min, G) 20 min. The right hind limbs were injected with Probe‐**2** only as the control group. H) Mean fluorescence intensities were recorded for the interested areas of control group, (1) left hind limbs and (2) right hind limbs. Values are the mean ± SD for each group of three experiments; *p < 0.05.

HOCl‐mediated inflammation and/or tissue destruction in RA in mice were then investigated using Probe‐**2** as a fluorescence probe. The RA was induced by the injection of λ‐carrageenan (5 mg mL^−1^, 100 µL in PBS) into the left hind limbs of 6–8 week old mice (Figure S29, Supporting Information). As the control group, the right hind limbs were injected with 100 µL PBS. After 4 h, the Probe‐**2** was injected into both the hind limbs of mice, and then images were recorded immediately after injection. As shown in **Figure** **6**, clear fluorescence emission from left ankles (1) were observed, where the λ‐carrageenan‐induced RA was being generated. Similar to the control group of healthy mice, negligible changes of fluorescence emission were observed for the right ankles where the PBS was injected. The results revealed that HOCl serves as an important mediator for RA in mice, and the endogenous HOCl generation in RA of mice could be visualized using Probe‐**2** as the fluorescence probe. Therefore, Probe‐**2** can be potentially used for RA diagnosing through monitoring of HOCl generation.

**Figure 7:**
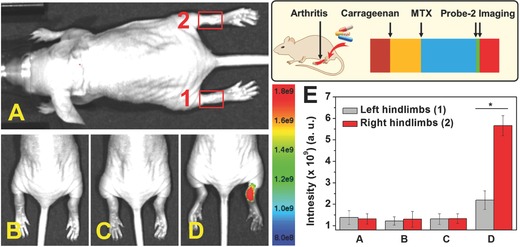
Monitoring of HOCl‐mediated RA treatment response. A) Control group; B) both left and right hind limbs were stimulated with λ‐carrageenan in PBS for 4 h; C) left hind limbs were administered with MTX for another 6 h; D) Probe‐**2** was injected locally into both right and left hind limbs. E) Mean fluorescence intensities were recorded for the interested areas of control group, (1) left hind limbs and (2) right hind limbs. Values are the mean ± SD for each group of three experiments; *p < 0.05.

For the treatment of RA, there is a pressing need for techniques for monitoring therapy response early in individual patients. In this context, we next evaluated the ability of Probe‐**2** for assessing the HOCl‐mediated RA treatment in living mice. RA at both left and right hind limbs were generated by λ‐carrageenan stimulation. MTX, a standard drug for RA patients, was administered to left hind limbs locally by injection. The control groups were injected with same amount of PBS. After 6 h treatment, imaging for both control group and treatment group were performed. As shown in **Figure** **7**, fluorescence signal from left hind limbs were significantly inhibited after MTX treatment, which is obviously different with the right hind limbs that PBS was administered. To the best of our knowledge, this is the first observation that inhibition of HOCl generation occurred during RA treatment by antiarthritic drug. Mean fluorescence intensity analysis showed around 2.5 times decrease in emission intensity, suggesting effective RA treatment by MTX. The results suggested that Probe‐**2** can be used as an effective fluorescence probe for early evaluation of RA treatment response through monitoring the changes of HOCl levels.

## 3 Conclusions

In conclusion, two new fluorescence probes, Probe‐**1** and Probe‐**2**, have been designed and synthesized for quantitative detection of HOCl‐mediated inflammatory disorders. The probes were easily synthesized, and the chemical structure and molecular weight were well characterized by HRMS, NMR, FTIR. In the presence of HOCl, both the probes showed significant changes in absorption and emission spectra as the result of the formation of **M_1_** and **M_2_** after cleavage of C=N bond. The superiority of the proposed probes, including high sensitivity and specificity, rapid fluorescence “OFF–ON” response, and biocompatibility enables real‐time monitoring of HOCl generation in vitro and in vivo. Using Probe‐**2** as the probe, microscopy imaging and flow cytometry analysis of HOCl‐mediated inflammatory response in cells were demonstrated, followed by visualizing LPS‐induced inflammation in living adult zebrafish. Importantly, the inflammatory response of RA in living mice and HOCl‐mediated RA treatment response were successfully demonstrated. We thus envision that the successful development of this probe can serve as a robust approach to contribute to future biomedical or clinical researches on the RA early diagnosis, treatment response monitoring, and the etiology studies of HOCl‐mediated inflammatory disorders.

## 4 Experimental Section

*Reagents and Instruments*: 3‐Hydroxy‐1,8‐naphthalic anhydride, hexamethylenetetramine (HMTA) were purchased from Aladdin reagent Co. (Shanghai, China). 2,3‐Diaminomaleonitrile, *n*‐butylamine, and trifluoroacetic acid were obtained from Sinopharm Chemical Reagent Co., Ltd. (China). 2,3‐Dihydroxybenealdehyde, metal ions (nitrate salts), anions (sodium salts) were purchased from Alfa Aesar. Hochest 33342, LPS, 3‐morpholinosydnonimine (SIN‐1) (ONOO^−^ donor), sodium hypochlorite (NaOCl), MTX, and λ‐carrageenan were purchased from Sigma‐Aldrich. Roswell Park Memorial Institute's Medium (RPMI‐1640), Dulbecco's modified Eagle medium (DMEM), fetal bovine serum (FBS), l‐glutamine, penicillin, streptomycin sulfate, trypsin‐EDTA (0.25%), PrestoBlue, MTT, and LysoSensor Green were purchased from Life Technologies (Australia). 1‐hydroxy‐2‐oxo‐3‐(3‐aminopropyl)‐3‐methyl‐1‐triazene (NOC‐13, NO donor) was synthesized following previously reported method.^27^ Nude mice (6–8 weeks) and zebrafish were obtained from the Experimental Animal Center of the Dalian Medical University, China. All the experiments of living nude mice and zebrafish were performed in compliance with the relevant local laws and institute guidelines, and also the institution committee of the Dalian Medical University had approved the experiments. Unless otherwise stated, solvents and reagents were of analytical grade from commercial suppliers and were used without further purification. Deionized water was used throughout.

^1^H‐NMR and ^13^C‐NMR spectra were recorded with an AVANCE600MHZ spectrometer (BRUKER) with chemical shifts reported as ppm (in DMSO, tetramethyl silane (TMS) as internal standard). High resolution mass spectra were recorded on an Agilent 6530 QTOF spectrometer. Fluorescence spectra were measured with Perkin Elmer LS55 luminescence spectrometer (USA). Absorption spectra were measured with a Perkin Elmer Lambda 900 UV/VIS/NIR spectrophotometer (USA). Quartz cuvettes with a 1 cm path length and 3 mL volume were involved in fluorescence and UV–vis spectrum measurements. The pH was recorded by OHAUS ST3100 digital pH‐meter. Fluorescent images were obtained using Leica SP8 laser‐scanning microscope. The excitation was performed at 488 nm, and emission of 560 ± 20 nm was collected. The images were analyzed by ImageJ software version 1.44p, and colocalization analysis was performed by a colocalization analysis plugin. Flow cytometry analysis was recorded on an Accuri C6 flow cytometer, BD Biosciences with a 488 nm laser excitation and emission 565 ± 20 nm. The data were analyzed with Flowjo software. Imaging of HOCl in mice was performed using a SPECTRAL Ami Imaging Systems (Spectral Instruments Imaging, LLC, Tucson, AZ) with an excitation filter 465 nm and an emission filter 610 nm. All the data were calculated using the region of interest (ROI) function of Amiview Analysis software (Version 1.7.06), and values were presented as the mean ± standard deviation (SD) for each group of three experiments.

*Synthesis and Characterization of Probes*: The synthesis procedure of Probe‐**1** and Probe‐**2** were illustrated in Scheme S1 (Supporting Information). The details of experiments were described as follows and in the Supporting Information.

*Synthesis and Characterization of Probes—Synthesis of Probe‐* ***1***: In a 100 mL round‐bottom flask, 2,3‐diaminomaleonitrile (0.445 g, 1.5 mmol) was dissolved in 15 mL methanol at 65 °C. Then, the solution of 2,3‐dihydroxybenzaldehyde (**M_1_**) (0.276 g, 2.0 mmol) in 25 mL methanol was added. The reaction mixture was then maintained at 65 °C for 6 h to form a yellow precipitate. After cooling down to room temperature, the crude product was filtered, washed with cooled methanol to obtain Probe‐**1** in 91% yield. ^1^H NMR (DMSO‐*d*, 600 MHz) δ (ppm): 9.64 (s, 2H), 8.58 (s, 1H), 7.83 (s, 2H), 7.46 (d, *J* = 7.9 Hz, 1H), 6.91 (s, *J* = 7.7 Hz, 1H), 6.71 (s, *J* = 7.8 Hz, 1H). ^13^C NMR (DMSO‐*d*, 150 MHz) δ (ppm): 154.3, 147.6, 146.2, 126.4, 122.2, 119.8, 119.7, 118.8, 115.1, 114.5, 103.9. Electrospray ionization (ESI)‐HRMS (*m*/*z*) Calcd for C_11_H_7_N_4_O_2_: 227.0574 [Probe‐**1**—H]^−^; Found: 227.0578. M.p.: 145.6–145.9 °C.

*Synthesis and Characterization of Probes—Synthesis of Probe‐* ***2***: 4‐formyl‐3‐hydroxy‐1,8‐naphthalic‐*n*‐butylimide (**M_2_**) was prepared according to the reported method.^28^ Probe‐**2** was synthesized following a similar method to the Probe‐**1**. Typically, in a 15 mL MeOH solution of 2,3‐diaminoaleodinitrile (0.445 g, 1.5 mmol) at 65 °C, the solution of **M_2_** (0.594 g, 2.0 × 10^−3^ m) in 25 mL MeOH was added. The mixture was heated to 65 °C for 6 h to form dark brown precipitate, which was then filtered and washed with cooled MeOH to obtain Probe‐**2** in 89% yield. ^1^H NMR (600 MHz, DMSO‐*d*): δ (ppm) 11.66 (s, 1H), 9.01 (d, *J* = 8.56 Hz, 1H), 8.84 (s, 1H), 8.12 (d, *J* = 7.12 Hz, 1H), 7.99 (s, 2H), 7.84 (s, 1H), 7.68 (t, 1H), 3.93 (t, 2H), 1.58 (m, 2H), 1.30 (m, 2H), 0.91 (t, 3H); ^13^CNMR (150 MHz, DMSO‐*d*): δ (ppm) 163.7, 162.8, 159.2, 158.4, 153.1, 149.0, 131.8, 130.5, 129.6, 127.0, 125.3, 122.8, 122.3, 117.1, 114.2, 104.6, 96.9, 30.0, 20.3, 14.2. ESI‐HRMS (*m*/*z*) Calcd for C_21_H_16_N_5_O_3_: 386.1259 [Probe‐**2**—H]^−^; Found: 386.1245. M.p.: 255.6–257.9 °C.

*Confocal Fluorescence Imaging of HOCl‐Mediated Inflammatory Mimic Macrophage Cells*: In confocal imaging of endogenous HOCl‐mediated inflammatory response in macrophage, J774A.1 cells in a 22 mm cover glass bottom culture dishes were initially incubated with the culture medium containing endotoxin (LPS) (1.0 µg mL^−1^) for 4 h. After washing with PBS (3 × 2 mL per dish), cells were then incubated with fresh culture medium containing Probe‐**2** (4 × 10^−6^ m) for 0.5 h. The cells were rinsed with PBS (3 × 2 mL per dish) and then subjected to luminescence imaging measurements on the confocal microscope. J774A.1 cells incubated with Probe‐**2** (5 × 10^−6^ m) for 0.5 h were employed as the control group. For intracellular HOCl colocalization analysis in macrophage, J774A.1 cells were further incubated with LysoSensor Green according to each protocol from Life Technologies.

*Fluorescence Imaging of HOCl in Nude Mice*: The nude mice (6–8 week old mice) were anesthetized by isoflurane in a flow of oxygen in during all of the experiments. For imaging of exogenous HOCl in living mice, Probe‐**2** (10 × 10^−6^ m, 125 µL) was injected into mice, followed by the injection of 10 µL HOCl at the concentration of 200 × 10^−6^ m in the same area. Imaging for the injection area was performed every 5 min within 30 min with an excitation filter 465 nm and an emission filter 610 nm.

For imaging of HOCl generation in RA of mice, λ‐carrageenan‐induced RA was first conducted. The left tibiotarsal joints (left hind limbs) of mice were first injected with 100 µL PBS solution containing λ‐carrageenan (5 mg mL^−1^). For the control group, the right tibiotarsal joints (right hind limbs) of the same mice were injected 100 µL PBS solution (no λ‐carrageenan). After 4 h, 125 µL Probe‐**2** (10 × 10^−6^ m) was administered by injection to the same area of both right and left joints, and then fluorescence imaging was performed in every 5 min within 20 min. Imaging of healthy mice was employed as the blank.

For assessing the treatment response toward MTX, RA was induced for both right and left hind limbs via a similar method described above. Then, the antiarthritic drug MTX was administered locally by injection (20 µg in 20 µL PBS). At 30 min, 1 h, 2 h, and 7 h after MTX administering, 125 µL Probe‐**2** (10 × 10^−6^ m) was administered by injection to the same area of both right and left joints, and then fluorescence imaging was performed after 5 min. Imaging of healthy mice was employed as the blank.

## Conflict of Interest

The authors declare no conflict of interest.

## Supporting information

Supplementary
