# Supplementary material for: Rapid Response Fluorescence Probe Enabled In Vivo Diagnosis and Assessing Treatment Response of Hypochlorous Acid‐Mediated Rheumatoid Arthritis
Source: Adv Sci (Weinh). 2018 Jun 7;5(8):1800397. doi: 10.1002/advs.201800397 (PMC6096987; doi:10.1002/advs.201800397)
Supplement: Supplementary file 1 — Supplementary [file ADVS-5-1800397-s001.pdf]

Copyright WILEY-VCH Verlag GmbH & Co. KGaA, 69469 Weinheim, Germany, 2018.

Supporting Information

for *Adv. Sci.,* DOI: 10.1002/advs.201800397

Rapid Response Fluorescence Probe Enabled In Vivo Diagnosis and Assessing Treatment Response of Hypochlorous Acid-Mediated Rheumatoid Arthritis

Huan Feng, Zhiqiang Zhang, Qingtao Meng, Hongmin Jia, Yue Wang, and Run Zhang**

Copyright WILEY-VCH Verlag GmbH & Co. KGaA, 69469 Weinheim, Germany, 2016.

Supporting Information

Rapid Response Fluorescence Probe Enabled in vivo Diagnosis and Assessing Treatment Response of Hypochlorous Acid Mediated Rheumatoid Arthritis

Huan Feng, Zhiqiang Zhang, Qingtao Meng, Hongmin Jia, Yue Wang, and Run Zhang**

School of Chemical Engineering, University of Science and Technology Liaoning, Anshan, Liaoning, 114051, P. R. China

E-mail: qtmeng@ustl.edu.cn, Tel.: +86-412-5929627

Australian Institute for Bioengineering and Nanotechnology, The University of Queensland, Brisbane, 4072, Australia

E-mail: r.zhang@uq.edu.au, Tel.: + 61 7 3346 3806, Fax: + 61 7 3346 3978

General information

Preparation of stock solutions of probes, ROS and anions

Stock solutions of Probe-**1** and Probe-**2** at the concentration of 0.5 mM were prepared by dissolving certain amount of probes in dimethyl sulfoxide (DMSO) and then kept in the dark at 4 ^{o}C. For analysis of HOCl in buffer, the stock solution was diluted into 20 mM phosphate-buffered saline (PBS) of pH 7.4 (DMSO: H_2O = 3:7) for all spectrometric measurements. Solutions of a series of anions and cations (20 mM) were freshly prepared by dissolving corresponding chemicals in deionized water. A stock solution of HOCl was prepared by dilution of the commercial sodium hypochlorite solution and stored according to the previous literatures.[1] The concentration of HOCl was determined by using its molar extinction coefficient of 391 M^{-1} cm^{-1} at 292 nm before use.[1] Hydroxyl radical (·OH) was generated in the Fenton system from ferrous ammonium sulfate and hydrogen peroxide.[2] Superoxide anion radical (O_2^{-}) was generated from the xanthine-xanthine oxidase system.[3] Singlet oxygen ($^1O_2$) was generated from the Na_2MoO_4-H_2O_2 system in 0.05 M carbonate buffer of pH 10.5.[4] $ONOO^{-}$ was donated by 3-morpholinosydnonimine (SIN-1).[5] Nitric oxide was generated by 1-hydroxy-2-oxo-3-(3-aminopropyl)-3-methyl-1-triazene (NOC-13).[6] Hydrogen peroxide (H_2O_2) was diluted immediately from a stabilized 30% solution, and was assayed using its molar absorption coefficient of 43.6 M^{-1} cm^{-1} at 240 nm.[7]

Quantum yield measurement

Fluorescence quantum yield was determined using optically matching solutions of fluorescein (Φ_f = 0.85 in 0.1 N NaOH aqueous solutions) as standard at an excitation wavelength of 470 nm and the quantum yield is calculated using the following equation (1).[8]

$$\Phi_{unk} = \Phi_{std} \frac{(F_{unk}/A_{unk})}{(F_{std}/A_{std})} \left(\frac{\eta_{unk}}{\eta_{std}} \right)^2$$

Where Φ_{unk} and Φ_{std} are the radiative quantum yields of the sample and standard, F_{unk} and F_{std} are the integrated emission intensities of the corrected spectra for the sample and standard, A_{unk} and A_{std} are the absorbance of the sample and standard at the excitation wavelength, and η_{unk} and η_{std} are the indices of refraction of the sample and standard solutions, respectively. Excitation and emission slit widths were modified to adjust the luminescent intensity in a suitable range. All the spectroscopic measurements were performed in triplicate and averaged.

Evaluation of partition coefficient

1-Octanol/water partition coefficients ($P_{o/w}$) for Probe-**2** was obtained using the 'shake-flask' method. Briefly, Probe-**2** was dissolved in water (1 mM), and was diluted with 2 mL 1-octanol-saturated PBS (pH 7.4). Then, 2 mL of water-saturated 1-octanol solution was added into the solution with continuous stirring. The mixture was stirred at R. T. for another 2 h before examining the concentrations of the complexes in water phase before and after partitioning by absorbance measurements. The $P_{o/w}$ of Probe-**2** was calculated according to the following formula: $P_{o/w} = (C_{before} – C_{after}) / C_{after}$. The experiment was repeated three times.

Cell line and cell culture

Human adenocarcinoma cell, HeLa (ATCC® CCL-2™) and mouse macrophage, J774A.1 (ATCC® TIB-67™) were obtained from American Type Cell Collection. HeLa cells were cultured in DMEM, supplemented with 10% FBS, 1% penicillin, 1% streptomycin sulphate in a humidified 5% CO_2/95% air incubator at 37 ^{o}C.J774A.1cells were maintained in RPMI 1640 medium, supported with 10% FBS, 100 U/mL penicillin, and 1% L-glutamine in a humidified 5% CO_2/95% air incubator at 37 ^{o}C. The growth medium was changed every two days. The HeLa cells were routinely subcultured trypsin-EDTA solution and growth to 80% confluence prior to experiments. For J774A.1 macrophage cells, cells were dislodged from flask substrate using a cell scraper, and then aspirated and dispense for subculture.

For cell experiments, stock solution of Probe-2 was prepared at the concentration of 4 mM in DMSO, and then diluted to the corresponding concentration used in the toxicity, flow cytometry, and imaging experiments with the DMSO concentration at 0.5%.

Cytotoxicity assay

*MTT assay of cytotoxicity of Probe-**2** in HeLa cells:* In this work, the cytotoxicity of Probe-**2** in live HeLa cells was investigated by MTT assay. HeLa cells were seeded at a density of 5 × 10^4 cells/mL in a 96-well micro-assay culture plate and growth for 24 h at 37 ^oC in a 5% CO_2/95% air incubator. The cell DMEM culture media of each well was then replaced with the fresh medium containing increasing concentration of Probe-**2**, *i.e.*, 0, 2, 5, 8, 10, and 20 μM. The wells with cell culture media only were employed as the blank. After incubation at 37 ^oC in a 5% CO_2/95% air incubator for 24 h, cell culture medium was removed and cells were washed three times with PBS. Then, 100 μL of 0.5 mg/mL MTT solution in PBS was added to each well, and the cells were incubated for another 4 h. The excess MTT solution was then carefully removed from each well, and the formed formazan was dissolved in 100 μL of DMSO (dimethyl sulfoxide). The optical density of each well was measured at a wavelength of 570 nm using a microplate reader (Bio-Tek, USA). The results from the five individual experiments were averaged. The following formula was used to calculate the viability of cell growth: Viability(%) = (mean of absorbance value of treatment group – blank)/(mean absorbance value of control – blank) × 100. All of the measurements were performed five times and the values are presented as the mean ± SD.

*PrestoBlue^{TM} Cell Viability assay of probe-**2** in J774A.1 macrophage:* The macrophage cells were typically seeded in a 96-well micro-assay culture plate at the density of 5000 cells/well. After 24 h incubation at 37 ^oC in a 5% CO_2/95% air incubator, the culture medium for each well was replaced with fresh medium containing Probe-**2** at the concentration of 0, 2, 5, 8, 10, and 20 μM, and incubated for another 24 h. Then the culture medium was replaced again with

100 μL RPMI/PrestoBlue™ (9/1, v/v), and incubated for another 10 min. Under excitation at 565 nm, the fluorescence intensity at 600 nm for each well was measured by microplate reader (Tecan X200). The fluorescence values of the no-cell control wells were measured as the blank, and the fluorescence intensity of the concentration of 0 μM were defined as the 100% viability. All of the measurements were performed five times and the values are presented as the mean ± SD.

Confocal fluorescence imaging of HOCl in HeLa cells

For visualizing exogenous HOCl in live HeLa cells, the cells were seeded at a density of 5 × 10^4 cells/mL in a 22 mm coverglass bottom culture dishes (ProSciTech, Australia). The cells were incubated at 37 °C in a 5% CO_2/95% air incubator for 24 h, before replacement of the culture medium with fresh DMEM containing 4 μM Probe-**2**. After 0.5 h incubation, the cells were washed with PBS (3×2 mL/dish), and then treated with 10 μM HOCl for another 15 min. The cells were washed with PBS for three times to remove excess HOCl and then subjected to confocal microscope fluorescence imaging.

Flow cytometry analysis of HOCl in HeLa cells

Flow cytometry analysis was employed for evaluating the "OFF-ON" fluorescence response of Probe-**2** towards HOCl. In a six-chamber culture well, the HeLa cells were seeded into each well at the density of 1×10^5 cells/mL. After 24 h, the ells were washed with PBS for three times, and then incubated with 4 μM Probe-**2** for 0.5 h. Then, the cells were further incubated with 10 μM HOCl for another 15 min. The cells were washed with PBS (3×2 mL/dish), detached from the well using trypsin-EDTA and then subjected to flow cytometry analysis.

Statistical analysis

The statistical significance of differences between the experimental group and control group was determined using one-way ANOVA. A p-value of less than 0.05 was considered to be significant (* means significantly different compared with the control group).

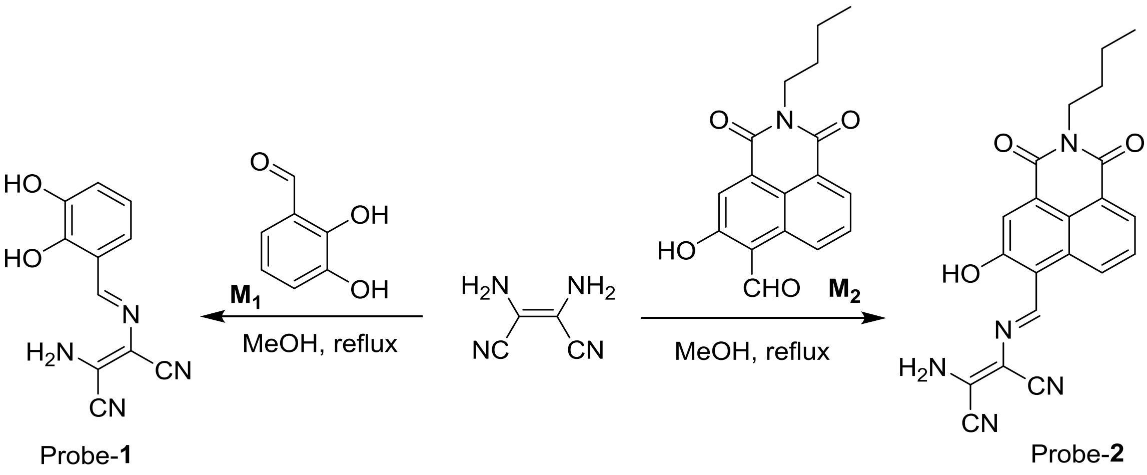

Scheme S1. Synthesis procedures of Probe-**1** and Probe-**2**.

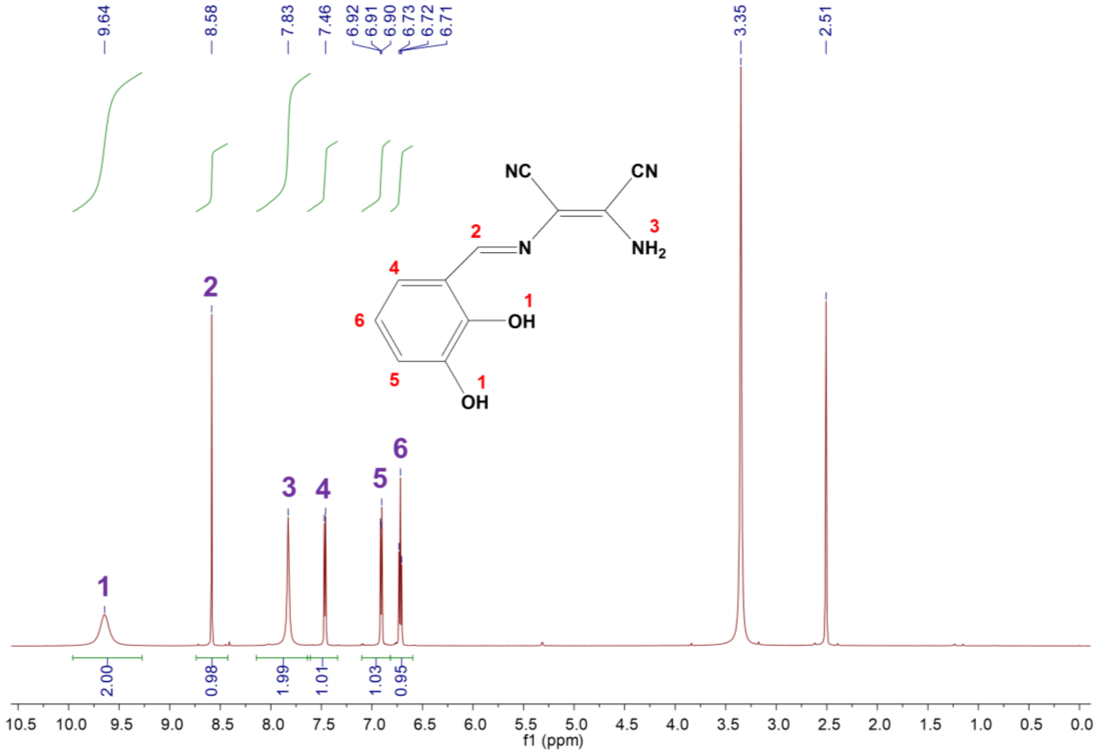

Figure S1. ^{1}H NMR spectrum of Probe-**1** (600 MHz, DMSO-d_6).

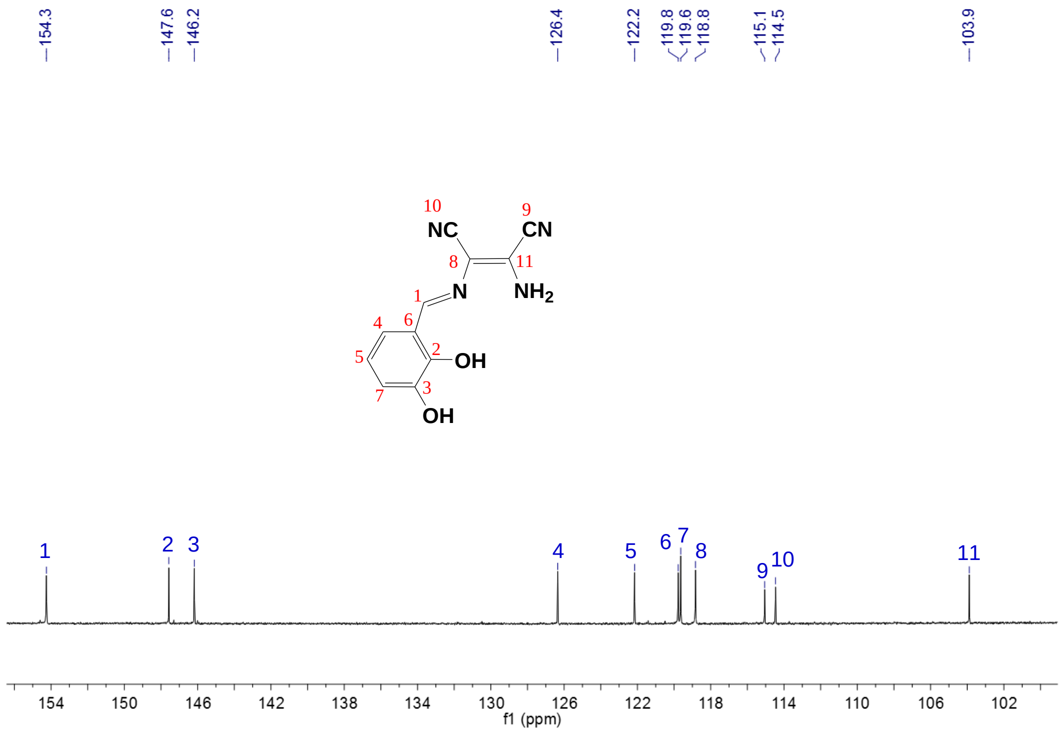

Figure S2. ^{13}C NMR spectrum of Probe-**1** (150 MHz, DMSO-d_6).

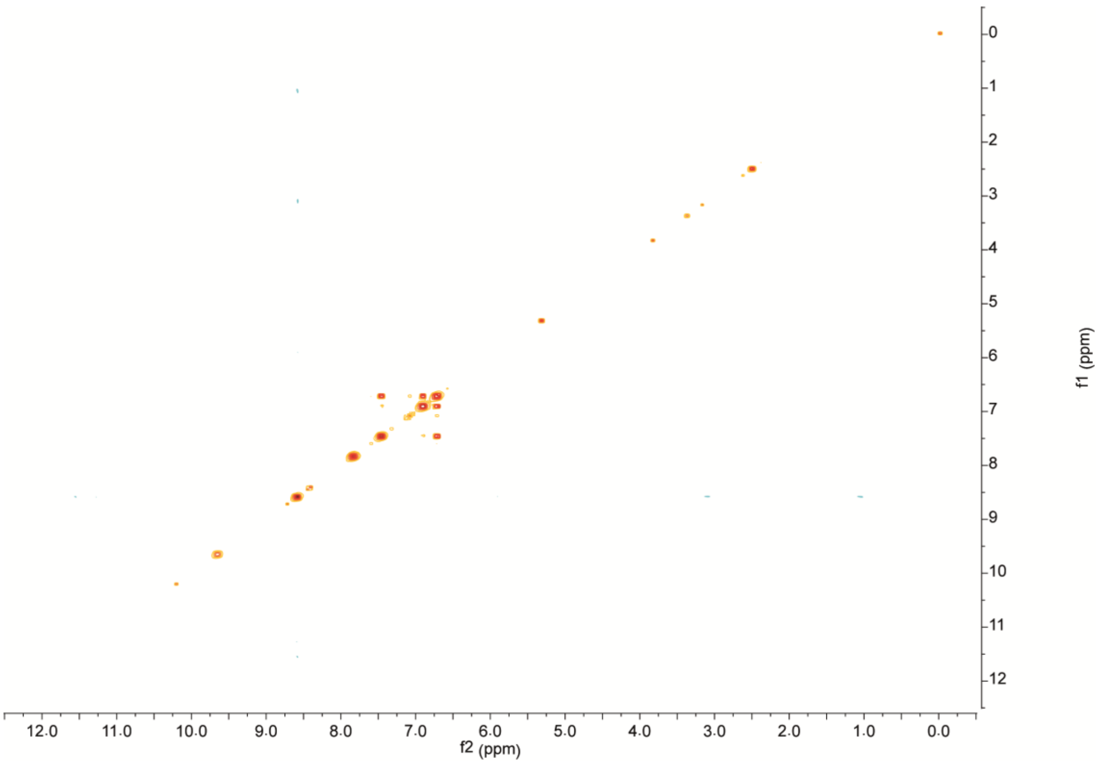

Figure S3. The ^1H-^1H 2D-COSY spectrum (400MHz NMR at 298 K) of Probe-**1**.

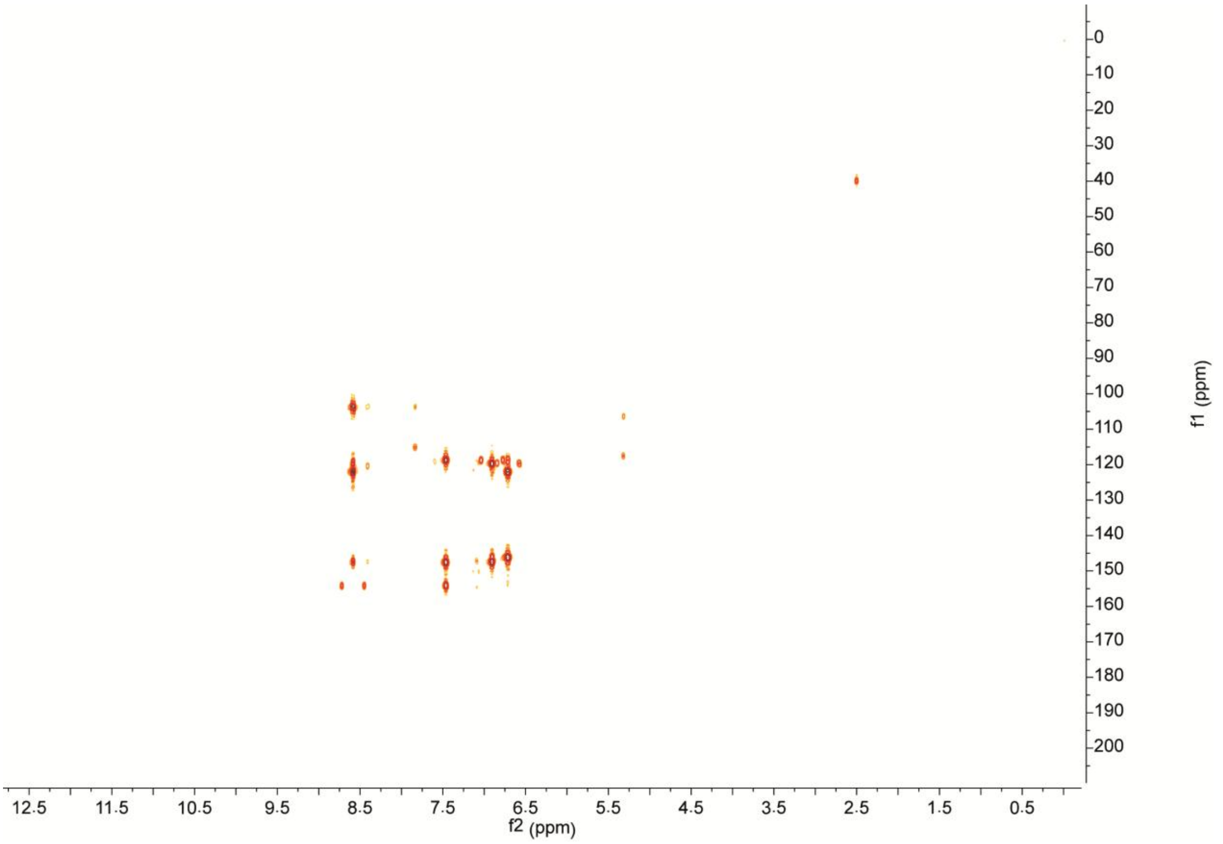

Figure S4. The ^{1}H-^{13}C 2D-HMBC spectrum (400MHz NMR at 298 K) of Probe-**1**.

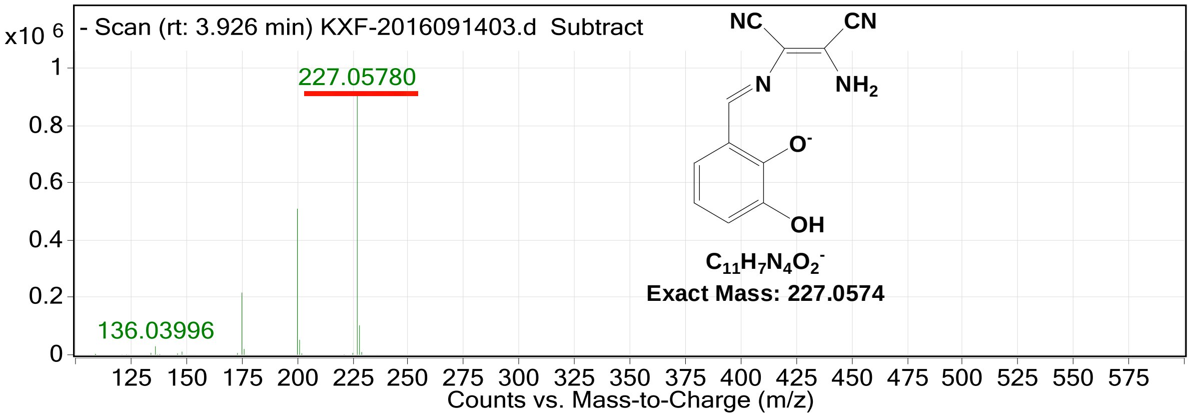

Figure S5. HR MS of Probe-**1**.

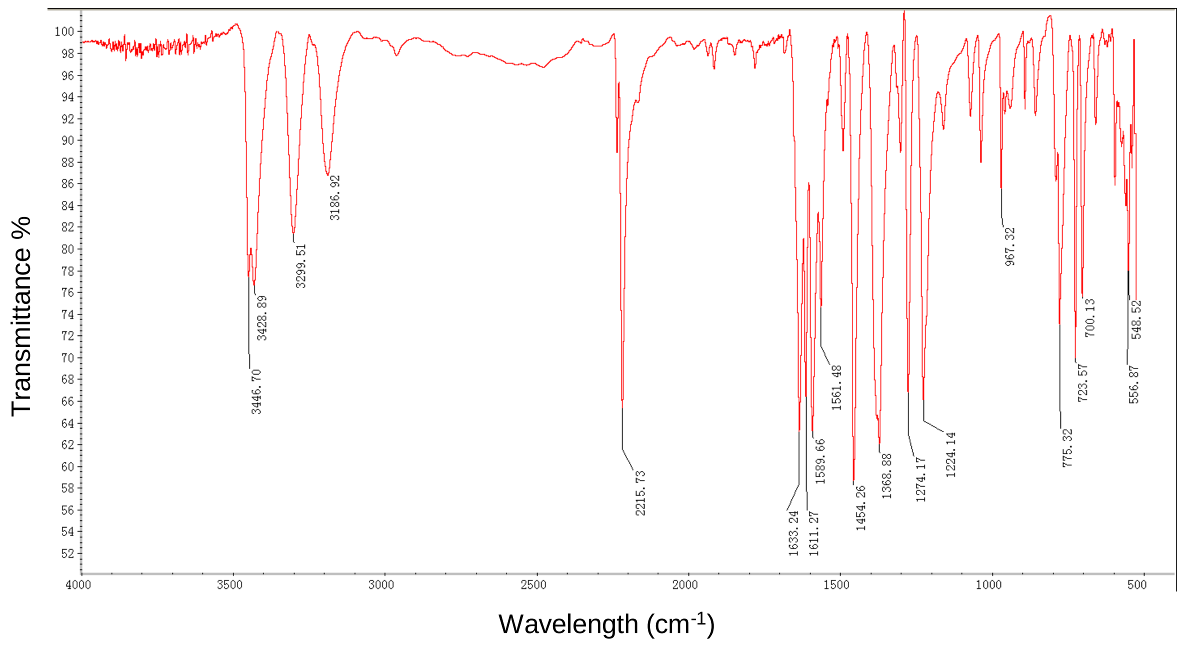

Figure S6. FTIR spectrum of Probe-**1**.

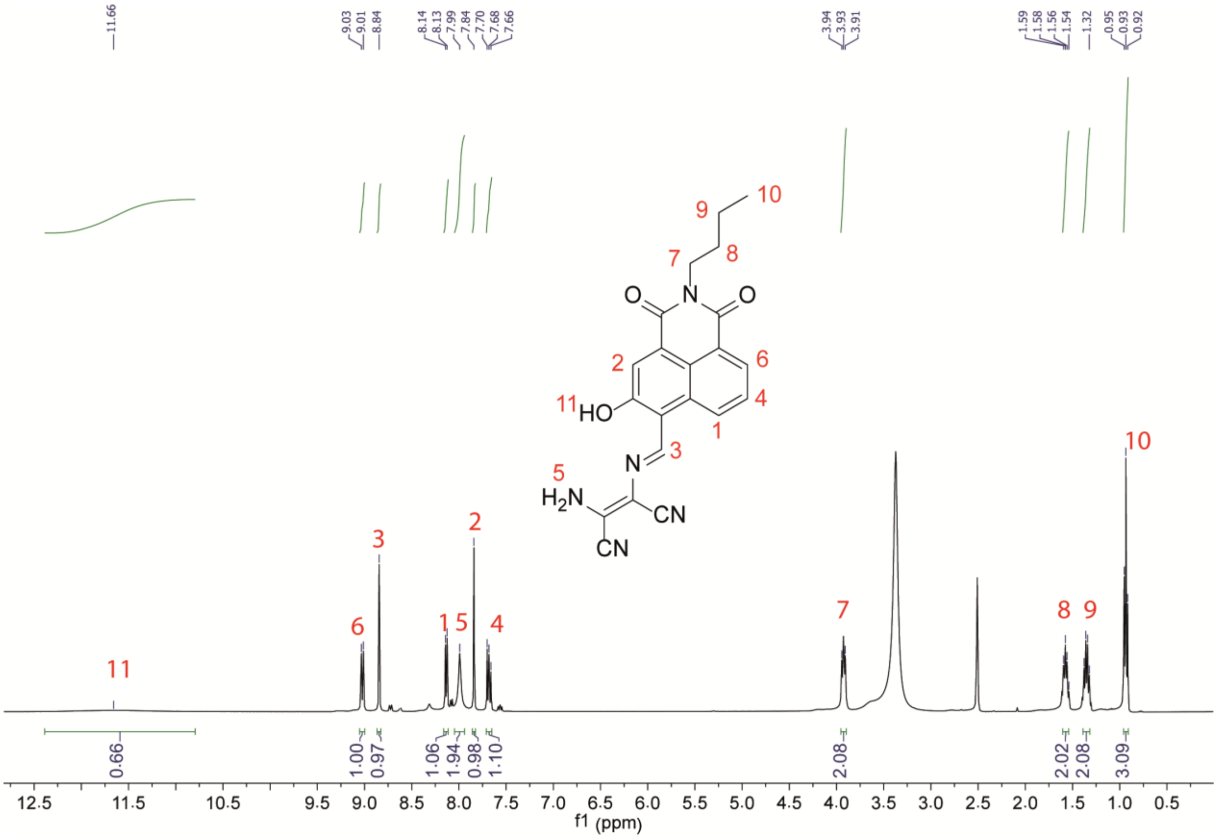

Figure S7. ^{1}H NMR spectrum Probe-**2** (600 MHz, DMSO-d_6).

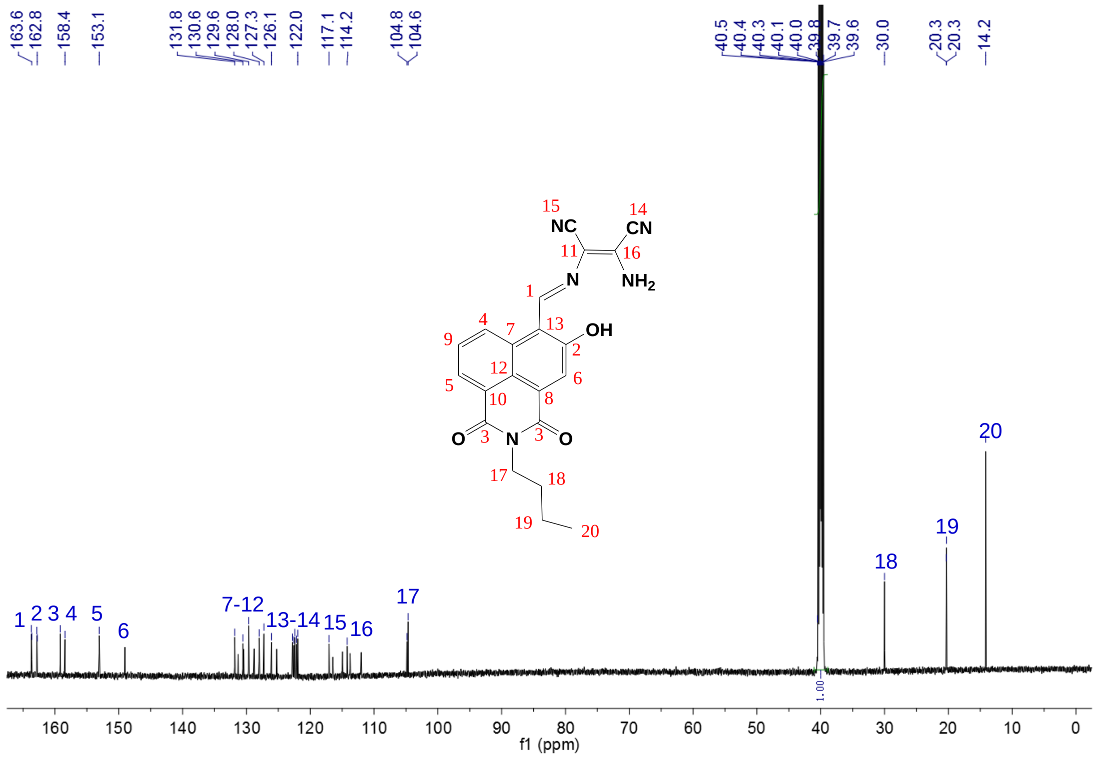

Figure S8. ^{13}C NMR spectrum of Probe-**2** (150 MHz, DMSO-d_6).

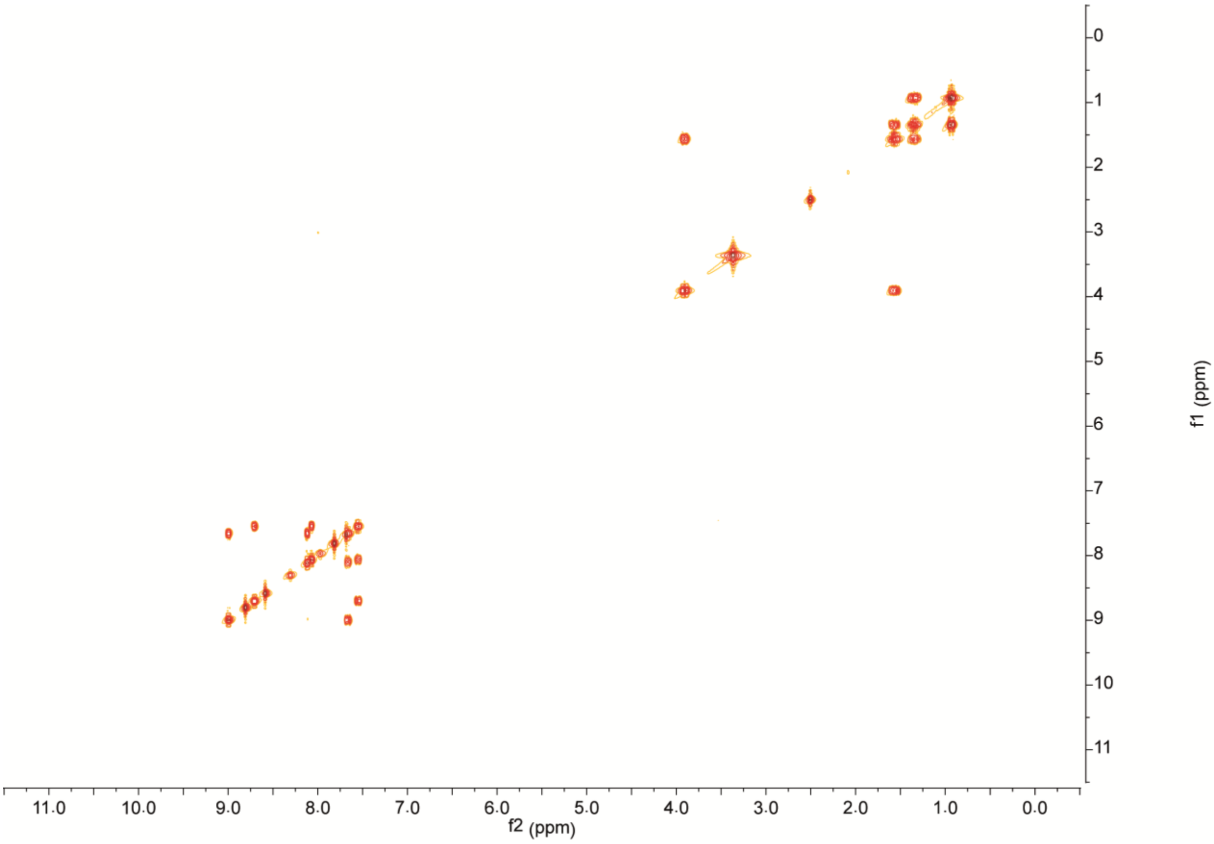

Figure S9. The ^{1}H-^{1}H 2D-COSY spectrum (400MHz NMR at 298 K) of Probe-**2**.

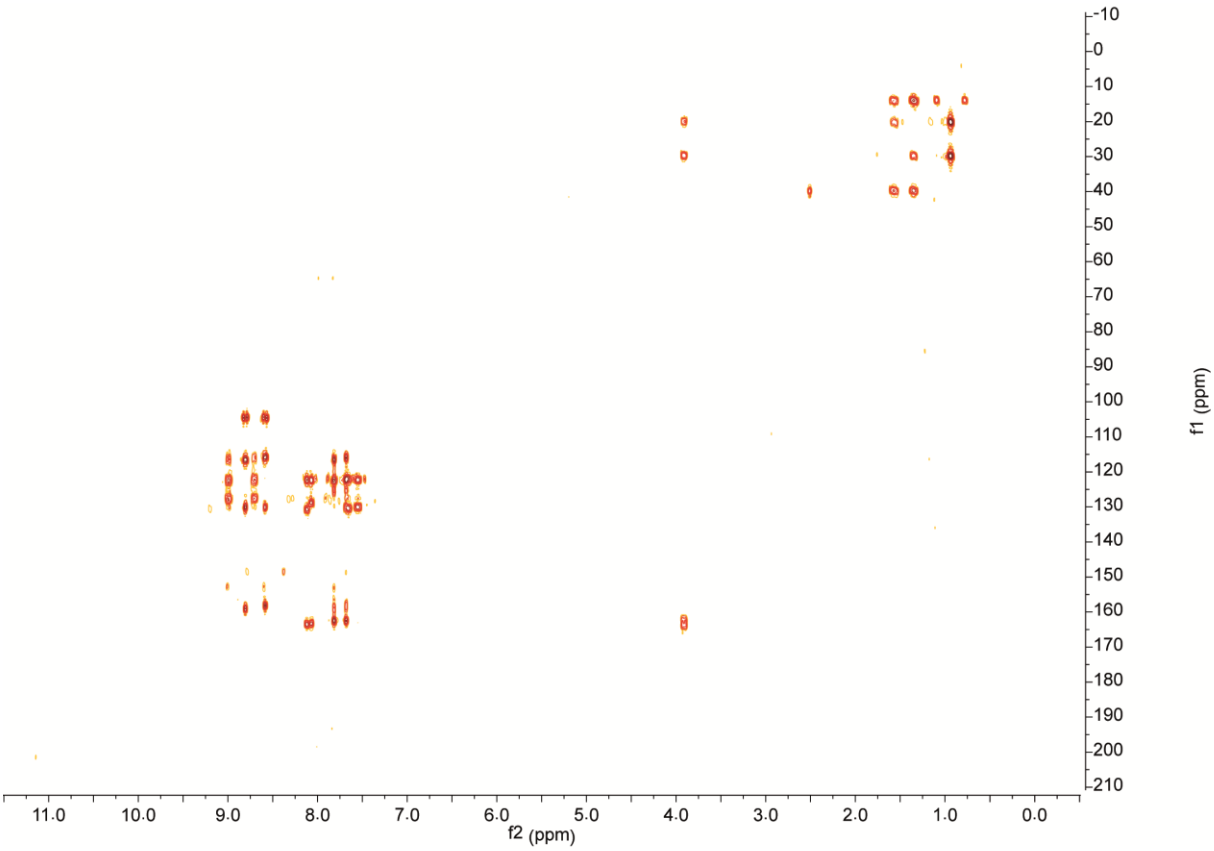

Figure S10. The ^{1}H-^{13}C 2D-HMBC spectrum (400MHz NMR at 298 K) of Probe-**2**.

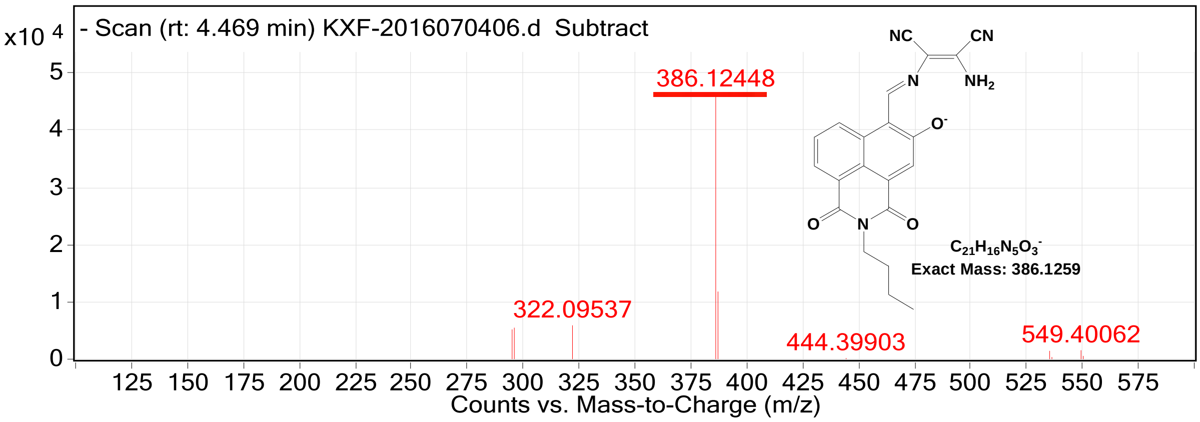

Figure S11. HR MS of Probe-**2**.

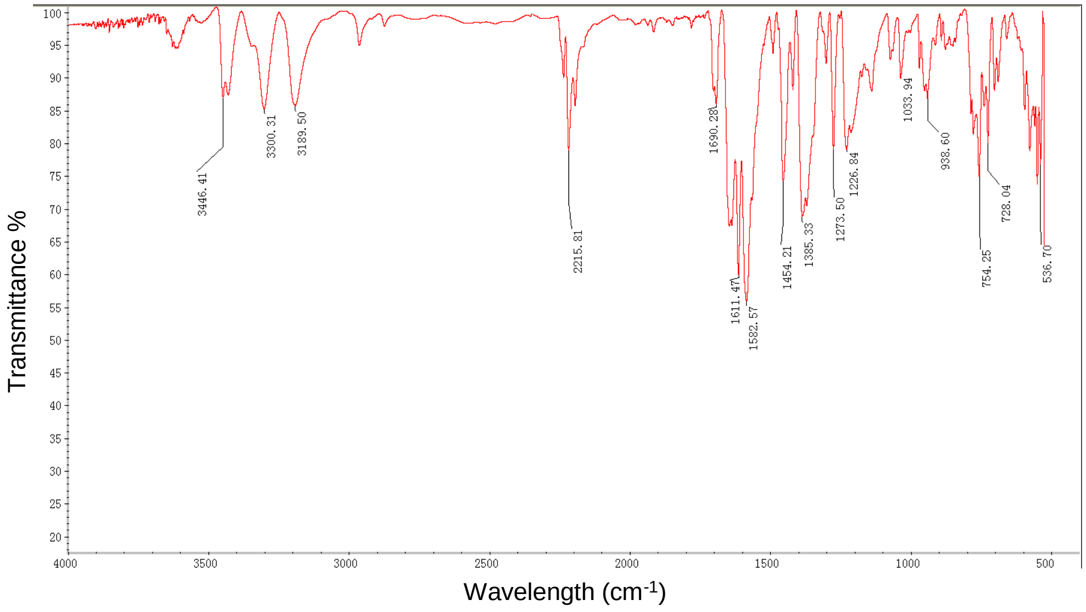

Figure S12. FTIR of Probe-**2**.

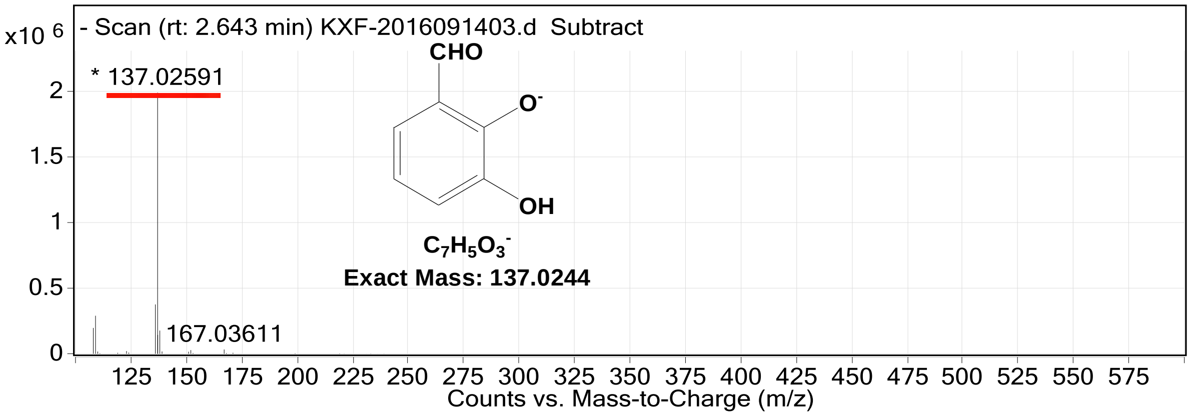

Figure S13. HR MS of Probe-**1** in the presence of HOCl.

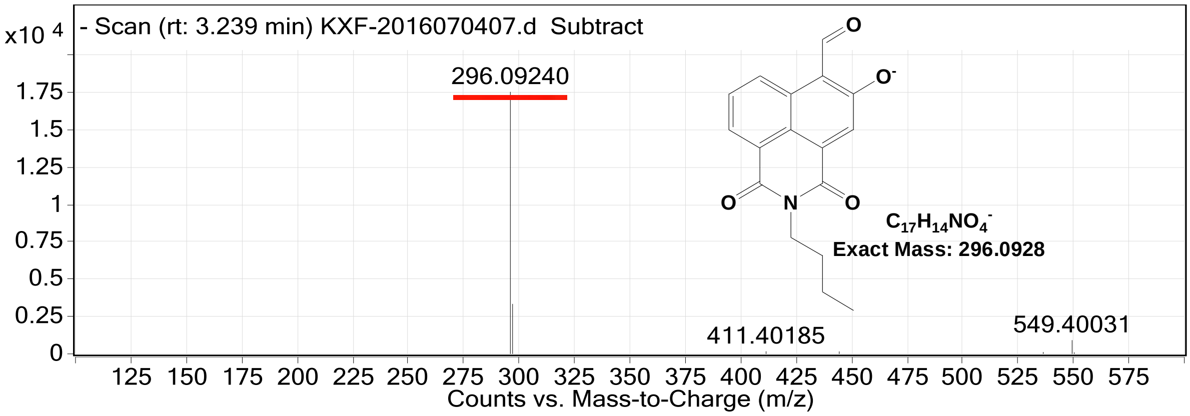

Figure S14. HR MS of Probe-**2** in the presence of HOCl.

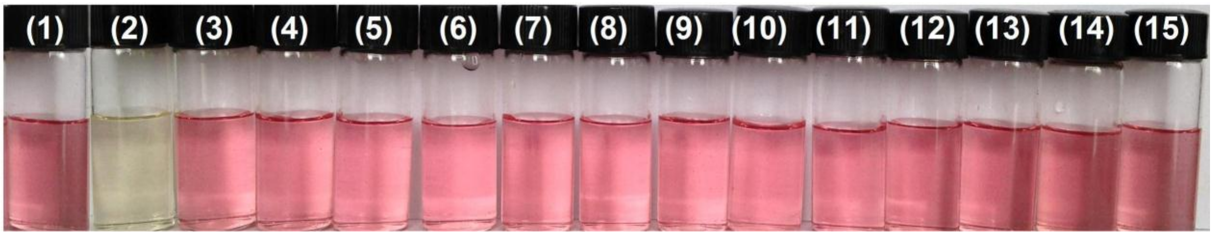

Figure S15. The colour changes of Probe-**2** (10 µM) upon addition of various ROS and anions species (100 µM) in PBS buffer (DMSO:H_2O=3:7, v/v; pH 7.4): (1) Probe-**2** only, (2) HOCl, (3) Cl^-, (4) Br^-, (5) I^-, (6) SO_4^{2-}, (7) HCO_3^-, (8) PO_4^{3-}, (9) Pi (10) NO_3^-, (11) NO_2^-, (12) $^1O_2$, (13) H_2O_2, (14) ·OH, (15) $ONOO^-$.

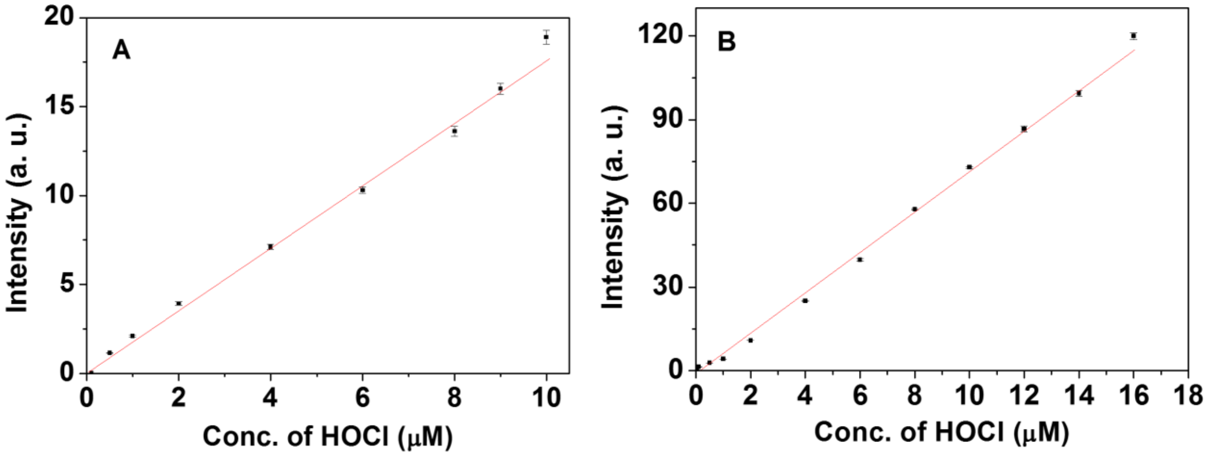

Figure S16. The linear calibration curves between the fluorescent intensity of Probe-**1** (2 µM), Probe-**2** (2 µM) and the concentrations of HOCl in PBS buffer (DMSO:H_2O=3:7, v/v; pH 7.4). Excitation at 370 nm and 490 nm for Probe-**1** and Probe-**2**, respectively.

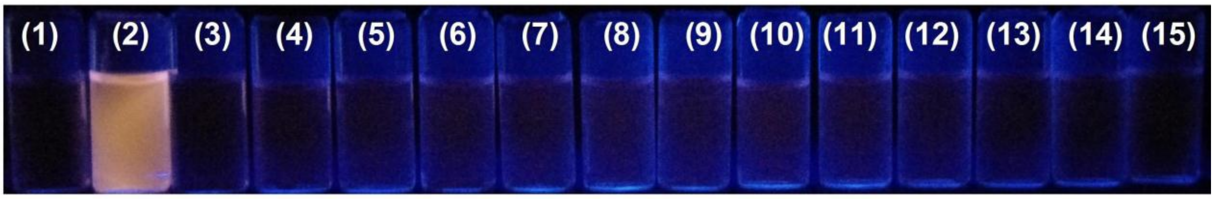

Figure S17. Fluorescence color responses of Probe-**2** towards various ROS and anions species in PBS buffer (DMSO:H_2O=3:7, v/v; pH 7.4). (1) Probe-**2** only, (2) HOCl, (3) Cl^-, (4) Br^-, (5) I^-, (6) SO_4^{2-}, (7) HCO_3^-, (8) PO_4^{3-}, (9) Pi (10) NO_3^-, (11) NO_2^-, (12) $^1O_2$, (13) H_2O_2, (14) ·OH, (15) $ONOO^-$.

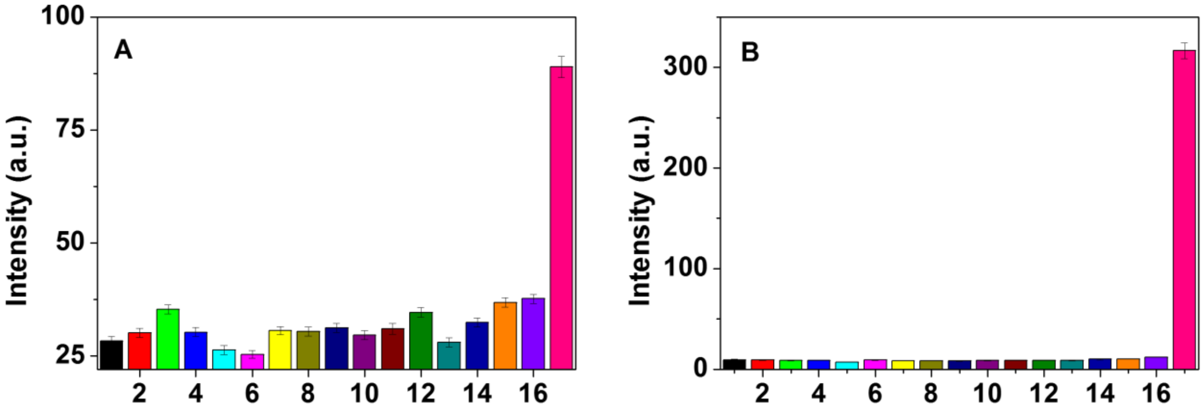

Figure S18. Fluorescence response of Probe-**1** (10 µM) and Probe-**2** (10 µM) towards various biological cations (150 µM) in PBS buffer (DMSO:H_2O=3:7, v/v; pH 7.4). The cations include 1. Li^+, 2. Na^+, 3. K^+, 4. Ca^{2+}, 5. Mg^{2+}, 6. Ba^{2+}, 7. Al^{3+}, 8. Fe^{3+}, 9. Cr^{3+}, 10. Zn^{2+}, 11. Co^{2+}, 12. Mn^{2+}, 13. Cu^{2+}, 14. Cys, 15. Hcy, 16. GSH, 17. HOCl. The excitation and emission wavelength are 370/427 nm, 490/575 nm for Probe-**1** and Probe-**2**, respectively.

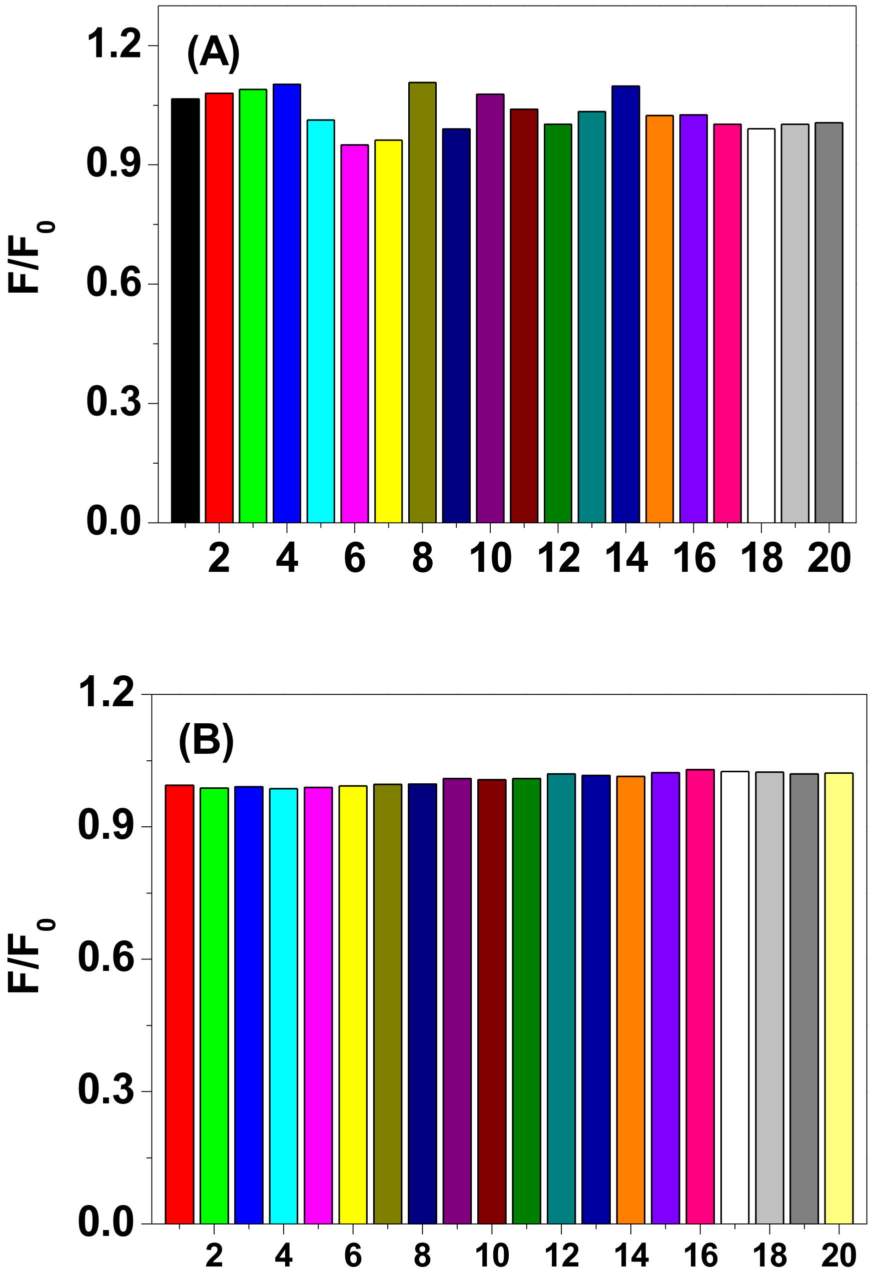

Figure S19. Changes of ffluorescence intensities of (A) $\mathbf{M_1}$ (10 μM), (B) $\mathbf{M_2}$ (10 μM) in PBS buffer (DMSO: H_2O = 3:7, v/v; pH 7.4) in the presence of various amino acids (100 μM): 1. Cys, 2. Met, 3.Ala, 4. Asp, 5. Asn, 6. Arg, 7. Gln, 8. Glu, 9. Gly, 10. His, 11. Ile, 12. Lys, 13. Leu, 14. Phe, 15. Pro, 16. Ser, 17. Thr, 18. Tyr, 19. Typ, 20. Val. Excitations at 370 for $\mathbf{M_1}$ and at 490 nm for $\mathbf{M_2}$.

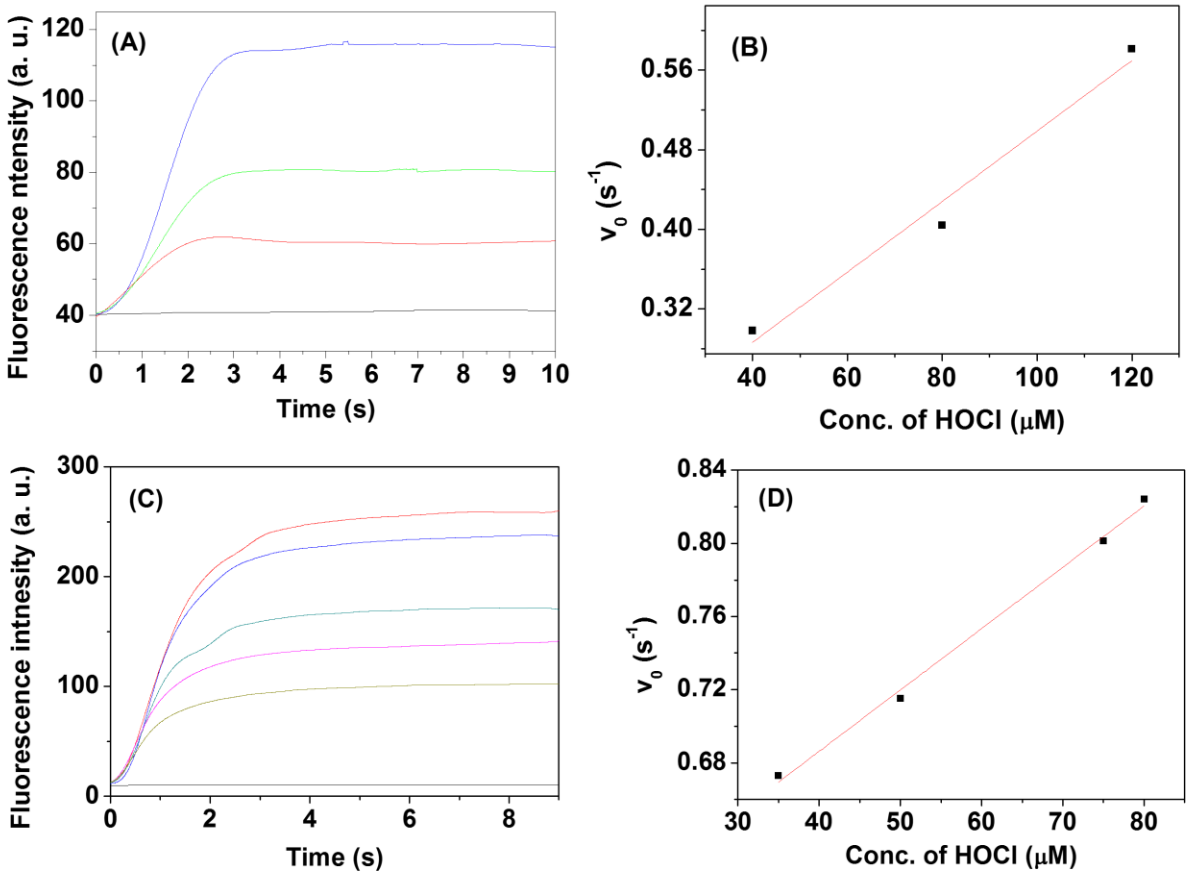

Figure S20. Reaction kinetic curves of (A) Probe-**1** (10 µM) and (C) Probe-**2** (10 µM) with different concentrations of HOC; the plot of initial rates (V_0) of the (A) Probe-**1** (10 µM) and (C) Probe-**2** (10 µM) reaction with HOCl versus their concentrations.

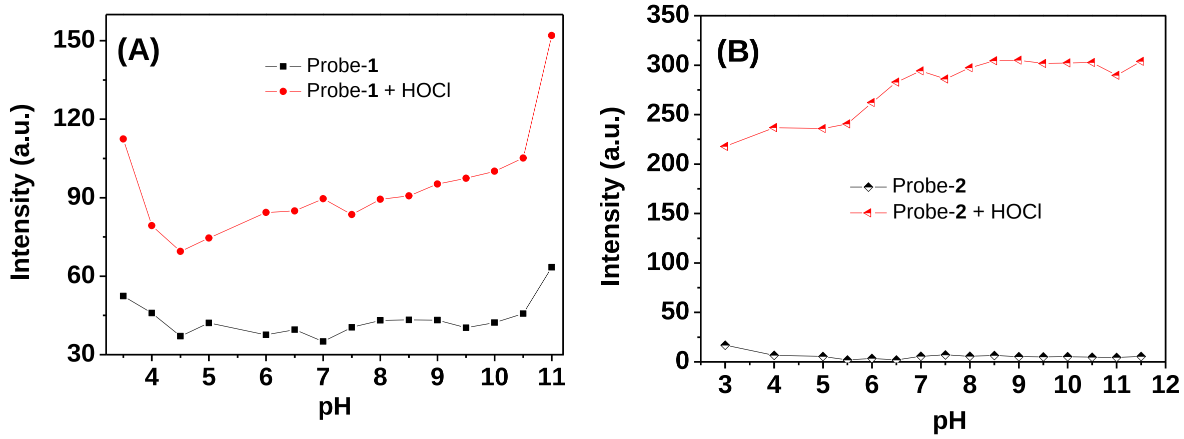

Figure S21. Effects of pH on the fluorescence intensity of Probe-**1** (10 μM) and Probe-**2** (10 μM) in the absence (black) and presence (red) of HOCl in PBS buffer (DMSO:H_2O=3:7, v/v). The excitation and emission wavelength ($\lambda_{ex}/\lambda_{em}$) are 370/427 nm, 490/575 nm for Probe-**1** and Probe-**2**, respectively.

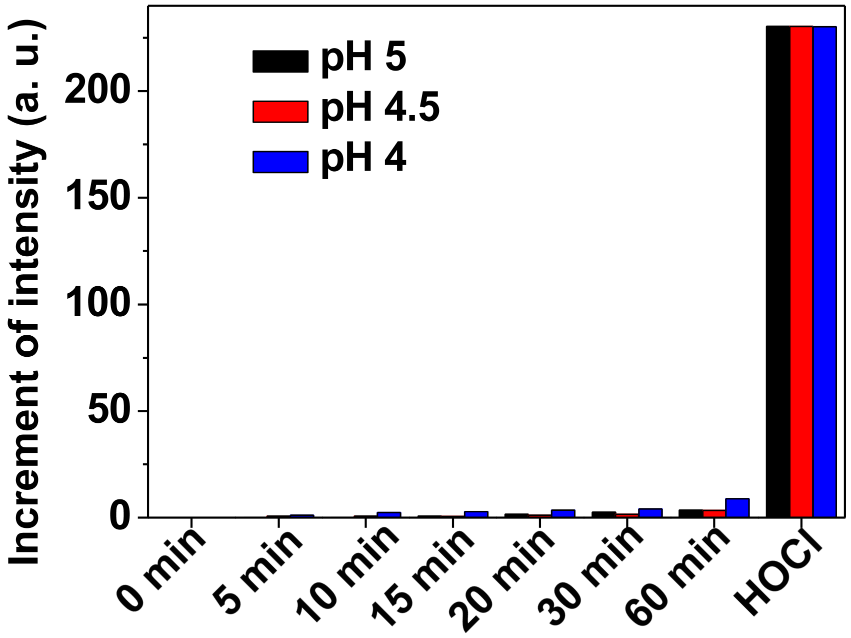

Figure S22. Changes of fluorescence intensity of Probe-**2** in pH 4, 4.5, 5 over the time. For comparison, HOCl was added into the solution of pH 4, 4.5, 5, and the fluorescence intensities were tested immediately after the addition.

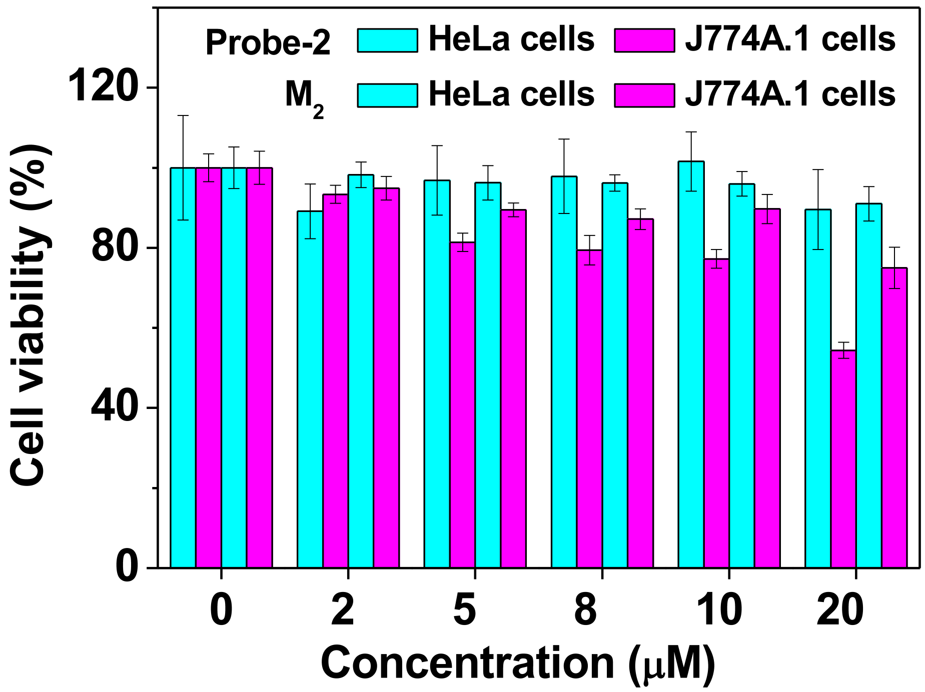

Figure S23. Cell viability values (%) of HeLa cells and J774A.1 macrophage cells treated with different concentration of Probe-**2** or $\mathbf{M_2}$ for 24 h.

The cytotoxicity of $\mathbf{M_2}$ towards HeLa cells and J774A.1 macrophage cells were then evaluated by MTT assay and PrestoBlue™ viability assay. HeLa cells were treated with $\mathbf{M_2}$ at the concentration of 0, 2, 5, 8, 10, 20 μM for 24 h, followed by the incubation with MTT for another 4 h. As shown in **Figure S19**, no obvious cytotoxicity of $\mathbf{M_2}$ to HeLa cells was obtained as the cell viability was more than 90% even after treated the HeLa cells with 20 μM $\mathbf{M_2}$ for 24 h. For the cytotoxicity of J774A.1 macrophage cells, the cells were firstly treated with $\mathbf{M_2}$ at the concentration of 0, 2, 5, 8, 10, 20 μM for 24 h, followed by the incubation with PrestoBlue™ for another 10 min. The results showed that the cell viability kept greater than 89% for the cells treated with 10 μM $\mathbf{M_2}$ for 24 h. When the concentration of the $\mathbf{M_2}$ increased to 20 μM, the cell viability was found to be more than 75% after 24 h co-incubation. These results indicated that the product of the reaction between Probe-**2** and HOCl, $\mathbf{M_2}$ is also low cytotoxicity.

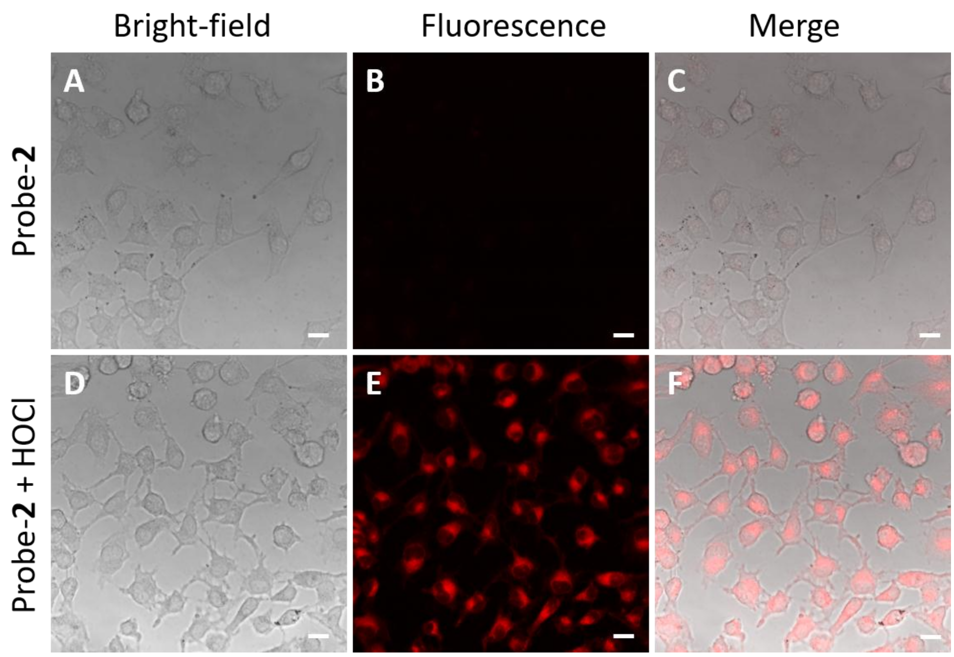

Figure S24. Bright-field, fluorescence, and merged images of HOCl in live HeLa cells. (A-C) the cells were incubated with 4 μM Probe-**2** for 30 min; (D-F) the Probe-**2** stained HeLa cells were then treated with 10 μM HOCl. Scale bar is 20 μM.

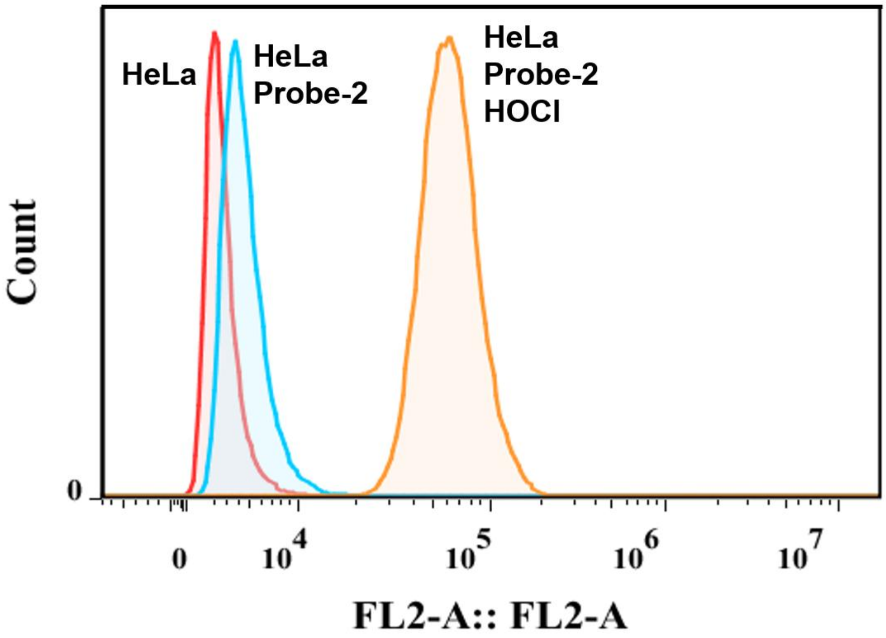

Figure S25. The shifts of histograms of flow cytometry analysis. HeLa cells were incubated with 4 μM Probe-**2** for 30 min, and then treated with 10 μM HOCl for 15 min.

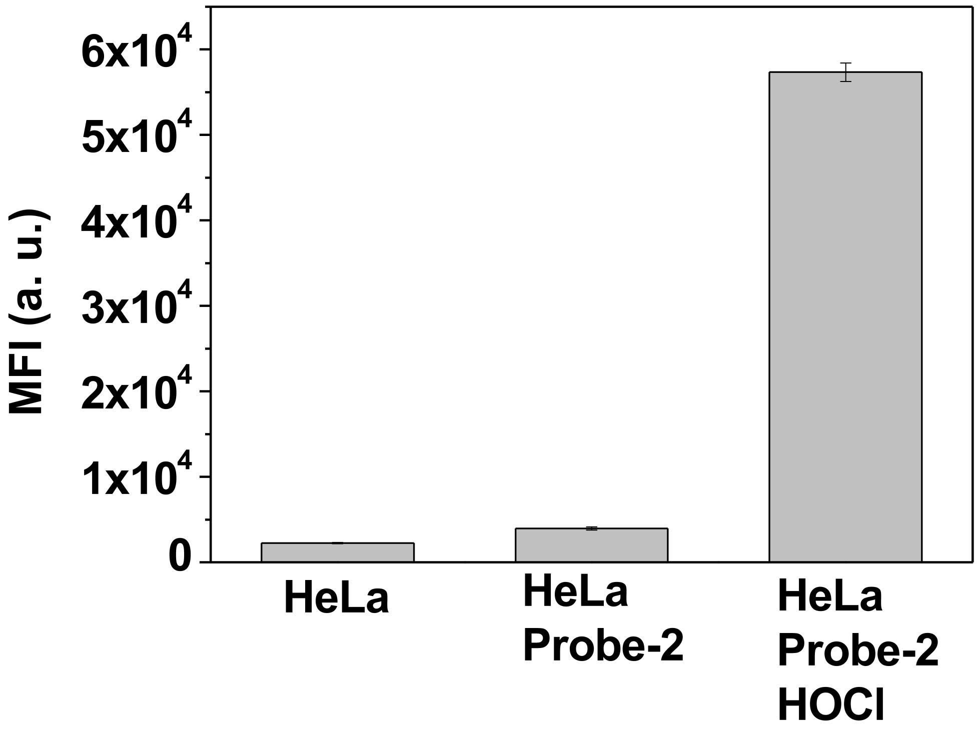

Figure S26. Mean fluorescence intensity per HeLa cell of different incubation conditions were examined by flow cytometry analysis.

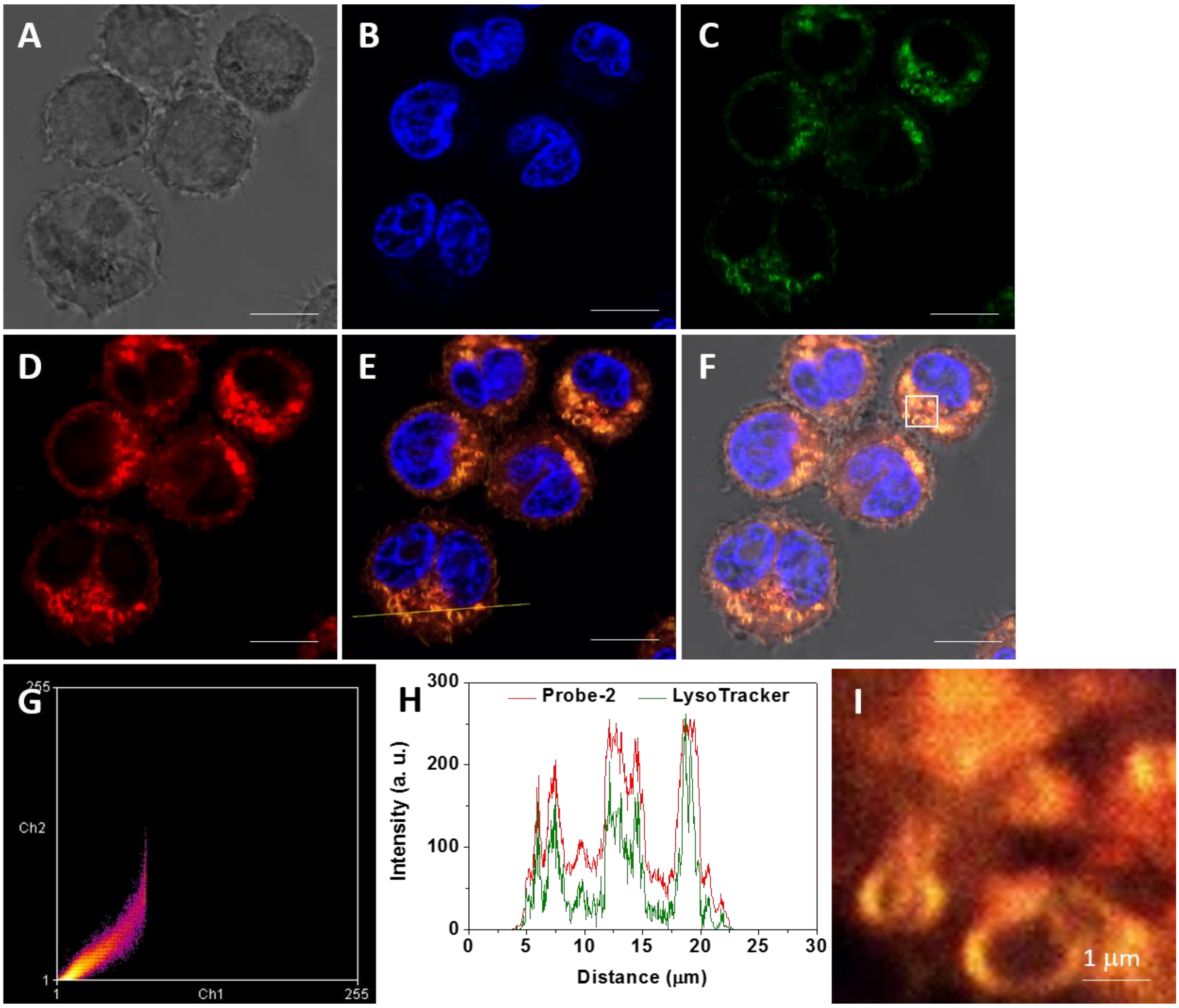

Figure S27. Intracellular co-localization analysis of Probe-**2** with LysoSensor™ Green in live J774A.1 macrophage cells. (A) Bright-field image of J774A.1 macrophage cells; (B) J774A.1 macrophage cell nucleus was stained with Hochest 33342; (C) the lysosome of J774A.1 cells was stained by LysoSensor™ Green; (D) cells were stimulated with LPS (1.0 μg/mL) for 4 h, and then incubated with Probe-**2** for another 30 min; (E) merged image of B, C, and D; (F) merged image of A and E; (G) intensity correlation plot of cells co-localized with Probe-**2** and LysoSensor™ Green; (H) fluorescence intensity profiles of the linear region of interest across macrophage cells in E; (I) magnified view of the region of interest in F. Scale bars are 20 μM.

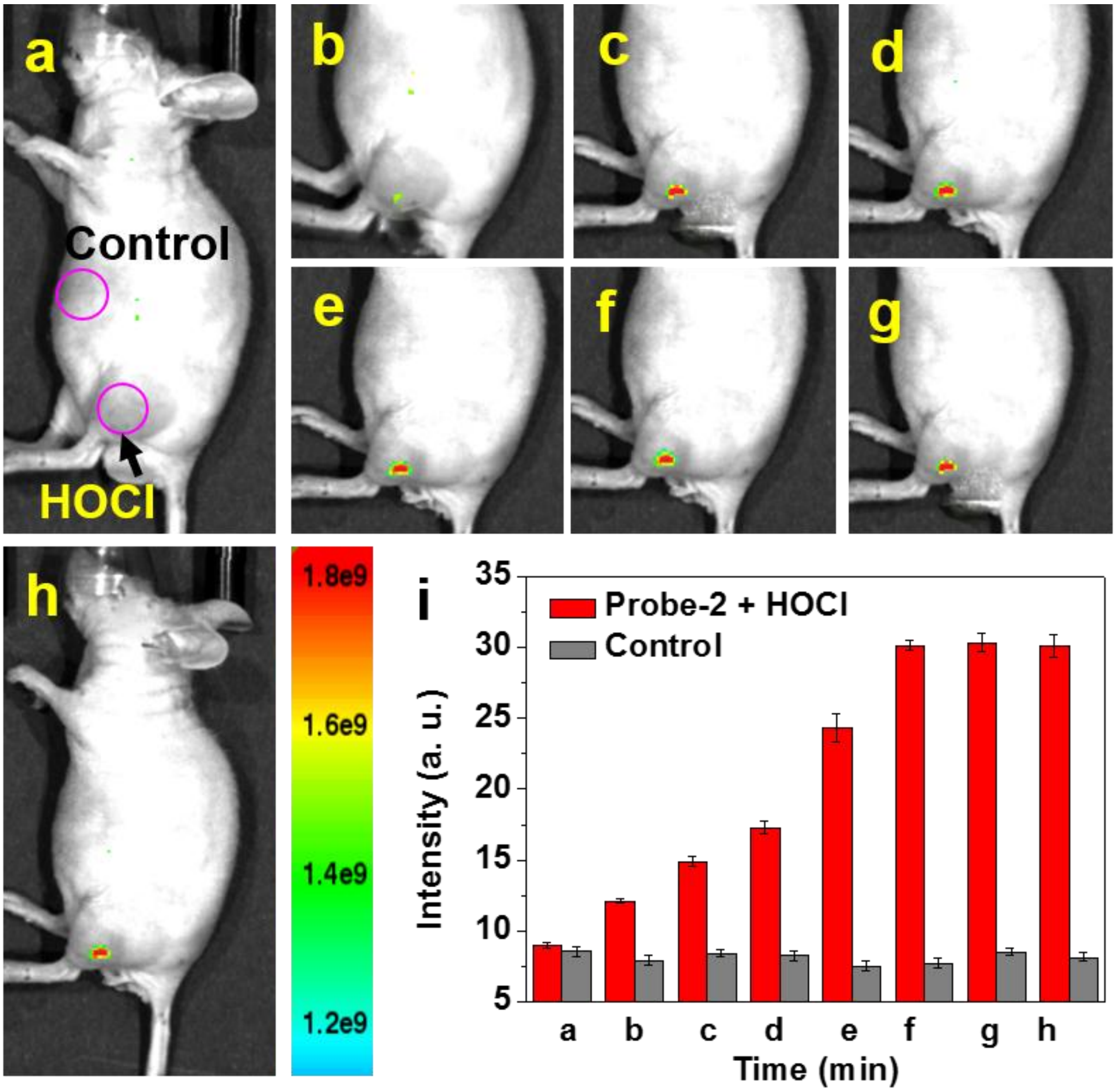

Figure S28. Fluorescence imaging of exogenous HOCl in live mice. (a) 125 μL Probe-**2** (100 μM) was subcutaneously injected into mice, followed by the injection of 10.0 μL HOCl (20 mM) to the area of interest (arrow in "a"). Images were recorded at different times, (b) 0 min; (c) 5 min; (d) 10 min; (e) 15 min; (f) 20 min; (g) 25 min; and (h) 30 min, respectively. The mean fluorescence intensities of areas of interest at different time showing in (a-h).

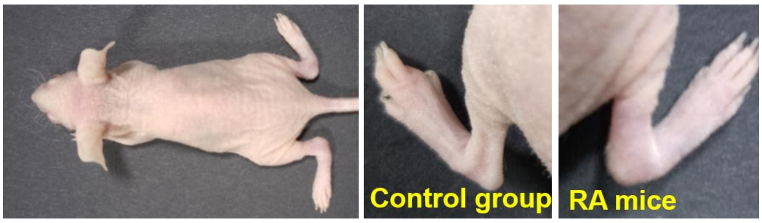

Figure S29. Images of arthritic hindlimbs of mice.

Table S1. Some recently reported fluorescent probes for HOCl detection.

Probes	Detection limit	Linearity range	Colour changes	Ex/Em (nm)	Response time	Intracellular analysis	In vivo/ex vivo sensing	Ref.
**RHHP**	1.40 nM	1-6 nM	Colourless-pink	520/580	< 30 s	Exogenous HOCl in HeLa cells	-	9
**R19-S**	-	-	-	515/585	-	Endogenous HOCl in neutrophils	(Ex vivo) intestinal HOCl in Drosophila melanogaster (endogenous HOCl)	10
**MMSiR**	-	0-5 μM	Colourless-blue	620/670	A few seconds	Endogenous HOCl in neutrophils	(In vivo) mouse peritonitis (endogenous HOCl)	11
**RSTPP**	9.00 nM	2-35 μM	Colourless-pink	553/580	2 min	Endogenous HOCl in mitochondria of macrophage (subcellular level)	-	12
**Rh-TPP**	0.11 μM	0-10 μM	Colourless-pink	540/575	-	Exogenous HOCl in mitochondria of HeLa cells (subcellular level)	(In vivo) nude mice (exogenous HOCl)	13
**BiTClO**	0.21 μM	0-20 μM	Colourless-pink	560/575	10 s	Exogenous HOCl in mitochondria of HeLa cells (subcellular level)	-	14
**Flu-1**	-	-	Colourless-yellow	454/530	-	Exogenous HOCl in HeLa cells	-	15
**FBS**	0.20μM	0-1 μM	-	498/523	-	-	(Ex vivo) intestinal HOCl in Drosophila melanogaster (endogenous HOCl)	16
**1**		0.5-100μM	-	480/542	1 min	Exogenous HOCl in HeLa cells and endogenous HOCl in macrophage (single cell level by flow cytometry analysis)	-	17
**MPhSeO-BOD**	-	0-40 μM	-	460/510	5 min	Endogenous HOCl in macrophage	-	18
**2**	4.5 nM	0-12 μM	-	492/507	A few	Endogenous	-	19

					seconds	HOCl in macrophage		
**B-Ts**	7.5 nM	0-15 μM	-	470/508	0.2 s	Exogenous HOCl in HeLa cells and endogenous HOCl in macrophage	-	20
**1b**	0.356 μM	0-20 μM	-	398/455	5 min	Exogenous HOCl in HepG2 cells	-	21
**HA**	0.7 μM	0-32 μM	-	340/466, 570	A few seconds	Exogenous HOCl in HeLa cells	-	22
**PZ-Py**	17.9 nM	0-80 μM	-	400/562	A few seconds	Exogenous HOCl in mitochondria HeLa, endogenous HOCl in mitochondria of RAW 264.7 (subcellular level)	(In vivo) nude mice (exogenous HOCl)	23
**Ptz-AO**	2.7 nM	0-0.5 μM	-	475/540	5 s	Exogenous HOCl in INS-1 β-islet cells and endogenous HOCl in macrophage	-	24
**Ru-Fc**	38.6 nM	2-15 μM	-	456/626	A few seconds	Exogenous/ endogenous in lysosome of MDA-MB-231, U-343 MGa, and macrophage cells	(In vivo) HOCl in Daphnia magna, zebrafish	25
**Ir-Fc**	93.3 nM	5-40 μM	-	400/600	< 1 s	Endogenous HOCl in mitochondria of Hep G2 cells	(In vivo) HOCl in liver injury of zebrafish; (ex vivo) HOCl in I/R liver of mice (two-photon and lifetime imaging)	26
**Probe-2**	17.3 nM	0-16 μM	Pink-yellow	490/575	< 4 s	Exogenous HOCl in HeLa cells and endogenous HOCl in lysosome of macrophage (single cell level by flow cytometry analysis, and subcellular level by imaging)	(In vivo) HOCl in nude mice (exogenous HOCl); HOCl in adult zebrafish, mice arthritis, and therapy of arthritis (endogenous HOCl generation and evolution in drug treatment).	This work

References

[1] S. Chen, J. Lu, C. Sun, H. Ma, *Analyst* **2010**, *135*, 577-582.

[2] K.-i. Setsukinai, Y. Urano, K. Kakinuma, H. J. Majima, T. Nagano, *J. Biol. Chem.*, **2003**, *278*, 3170-3175.

[3] H. Maeda, K. Yamamoto, Y. Nomura, I. Kohno, L. Hafsi, N. Ueda, S. Yoshida, M. Fukuda, Y. Fukuyasu, Y. Yamauchi, N. Itoh, *J. Am. Chem. Soc.*, **2005**, *127*, 68-69.

[4] B. Song, G. Wang, M. Tan, J. Yuan, *J. Am. Chem. Soc.*, **2006**, *128*, 13442-13450.

[5] a) J. J. Hu, N.-K. Wong, S. Ye, X. Chen, M.-Y. Lu, A. Q. Zhao, Y. Guo, A. C.-H. Ma, A. Y.-H. Leung, J. Shen, D. Yang, *J. Am. Chem. Soc.*, **2015**, *137*, 6837-6843; b) H. Li, X. Li, X. Wu, W. Shi, H. Ma, *Anal. Chem.*, **2017**, *89*, 5519-5525; c) J. Li, C. S. Lim, G. Kim, H. M. Kim, J. Yoon, *Anal. Chem.*, **2017**, *89*, 8496-8500.

[6] R. Zhang, Z. Ye, G. Wang, W. Zhang, J. Yuan, *Chem. Eur. J.*, **2010**, *16*, 6884-6891.

[7] V. Carroll, B. W. Michel, J. Blecha, H. VanBrocklin, K. Keshari, D. Wilson, C. J. Chang, *J. Am. Chem. Soc.*, **2014**, *136*, 14742-14745.

[8] Q. Meng, R. Zhang, H. Jia, X. Gao, C. Wang, Y. Shi, A. V. Everest-Dass, Z. Zhang, *Talanta* **2015**, *143*, 294-301.

(9) Goswami, S.; Das, A. K.; Manna, A.; Maity, A. K.; Saha, P.; Quah, C. K.; Fun, H.-K.; Abdel-Aziz, H. A. *Anal. Chem.*, **2014**, *86*, 6315-6322.

(10) Chen, X.; Lee, K.-A.; Ha, E.-M.; Lee, K. M.; Seo, Y. Y.; Choi, H. K.; Kim, H. N.; Kim, M. J.; Cho, C.-S.; Lee, S. Y.; Lee, W.-J.; Yoon, J. *Chem. Commun.*, **2011**, *47*, 4373-4375.

(11) Koide, Y.; Urano, Y.; Hanaoka, K.; Terai, T.; Nagano, T. *J. Am. Chem. Soc.*, **2011**, *133*, 5680-5682.

(12) Zhou, J.; Li, L.; Shi, W.; Gao, X.; Li, X.; Ma, H. *Chem. Sci.*, **2015**, *6*, 4884-4888.

(13) Hou, J.-T.; Wu, M.-Y.; Li, K.; Yang, J.; Yu, K.-K.; Xie, Y.-M.; Yu, X.-Q. *Chem. Commun.*, **2014**, *50*, 8640-8643.

(14) Li, K.; Hou, J.-T.; Yang, J.; Yu, X.-Q. *Chem. Commun.*, **2017**, *53*, 5539-5541.

(15) Cheng, X.; Jia, H.; Long, T.; Feng, J.; Qin, J.; Li, Z. *Chem. Commun.*, **2011**, *47*, 11978-11980.

(16) Xu, Q.; Lee, K.-A.; Lee, S.; Lee, K. M.; Lee, W.-J.; Yoon, J. *J. Am. Chem. Soc.*, **2013**, *135*, 9944-9949.

(17) Zhan, Z.; Liu, R.; Chai, L.; Li, Q.; Zhang, K.; Lv, Y. *Anal. Chem.*, **2017**, *89*, 9544-9551.

(18) Wang, B.; Li, P.; Yu, F.; Song, P.; Sun, X.; Yang, S.; Lou, Z.; Han, K. *Chem. Commun.*, **2013**, *49*, 1014-1016.

(19) Mulay, S. V.; Choi, M.; Jang, Y. J.; Kim, Y.; Jon, S.; Churchill, D. G. *Chem. Eur. J.*, **2016**, *22*, 9642-9648.

(20) Qiao, L.; Nie, H.; Wu, Y.; Xin, F.; Gao, C.; Jing, J.; Zhang, X. *J. Mater. Chem. B* **2017**, *5*, 525-530.

(21) Long, L.; Wu, Y.; Wang, L.; Gong, A.; Hu, F.; Zhang, C. *Chem. Commun.*, **2015**, *51*, 10435-10438.

(22) Guo, T.; Cui, L.; Shen, J.; Wang, R.; Zhu, W.; Xu, Y.; Qian, X. *Chem. Commun.*, **2013**, *49*, 1862-1864.

(23) Xiao, H.; Xin, K.; Dou, H.; Yin, G.; Quan, Y.; Wang, R. *Chem. Commun.*, **2015**, *51*, 1442-1445.

(24) Liang, L.; Liu, C.; Jiao, X.; Zhao, L.; Zeng, X. *Chem. Commun.*, **2016**, *52*, 7982-7985.

(25) Cao, L.; Zhang, R.; Zhang, W.; Du, Z.; Liu, C.; Ye, Z.; Song, B.; Yuan, J. *Biomaterials* **2015**, *68*, 21-31.

(26) Zhang, F.; Liang, X.; Zhang, W.; Wang, Y.-L.; Wang, H.; Mohammed, Y. H.; Song, B.; Zhang, R.; Yuan, J. *Biosens. Bioelectron.*, **2017**, *87*, 1005-1011.
